# The impact of plant-rich diets on sleep: a mini-review

**DOI:** 10.3389/fnut.2024.1239580

**Published:** 2024-02-06

**Authors:** Anastasiia Polianovskaia, Michelle Jonelis, Joseph Cheung

**Affiliations:** ^1^Department of Allergy, Pulmonary and Sleep Medicine, Mayo Clinic Jacksonville, Jacksonville, FL, United States; ^2^Sleep and Lifestyle Medicine Physician, Lifestyle Sleep, Mill Valley, CA, United States; ^3^Department of Allergy, Pulmonary and Sleep Medicine, Mayo Clinic Jacksonville, Jacksonville, FL, United States

**Keywords:** a plant-rich diet, plant-based diet, dietary Fiber, saturated fat, high-fat diet, sleep, sleep quality, obstructive sleep apnea

## Abstract

Plant-rich diets (PRDs), also referred to as plant based diets, have been shown to have beneficial effects on various chronic diseases and all-cause mortality. However, limited data are available on the effect of such diets on sleep and sleep disorders. In this review article, we explore existing evidence and potential mechanisms by which PRDs may impact sleep and sleepiness. High-fat diets are associated with drowsiness, while fiber-rich diets improve sleep quality. Anti-inflammatory diets may benefit patients with sleep disturbances, and diets rich in tryptophan and serotonin precursors may improve sleep quality. Isoflavones and polyphenols present in PRDs may also have a positive impact on sleep. Furthermore, diets rich in plants may reduce the risk of obstructive sleep apnea and associated daytime sleepiness. Overall, the current knowledge about PRDs in sleep and sleep disorders is limited, and further research is needed to explore the potential advantages of this dietary approach in sleep disorders.

## Introduction

Plant-rich diets (PRDs), also referred to as plant based diets, are known to be beneficial for overall health. In particular, eating high levels of fiber-rich, minimally processed fruits, vegetables, legumes, and whole grains while minimizing the intake of highly processed foods and animal products seems particularly beneficial. For instance, a prospective cohort study with 135,335 participants from 18 countries showed that consuming more fruits, vegetables, and legumes is linked to a reduced risk of noncardiovascular and total mortality. The most notable benefits for both noncardiovascular and total mortality were observed at three to four servings per day (375–500 g/day) of fruits, vegetables, and legumes (1). Additionally, the results of a systematic review of 32 prospective cohort studies and a meta-analysis of 31 studies of 715,128 participants showed a significant inverse dose–response association between plant protein intake and all-cause mortality (2). It concluded that plant protein consumption has been associated with a decreased risk of all-cause mortality and cardiovascular disease. Substituting animal protein sources with plant-rich protein sources may promote longevity. In 2016, a meta-analysis of 45 studies provided further evidence; that consuming whole grains is associated with a lower risk of coronary heart disease, cardiovascular disease, total cancer, all-cause mortality, respiratory illnesses, infectious diseases, diabetes, and all other noncardiovascular, noncancer causes (3).

Despite the ever-increasing amount of research supporting PRDs to reduce chronic illness and improve overall health, there is a paucity of research on the impact of these diets on sleep health. This review explores the effects of PRDs on sleep and sleepiness while also exploring the potential mechanism of these effects. The evidence presented encompasses several aspects, including dietary fiber, melatonin precursors, polyphenols, modulation of the gut microbiome, and their implications for sleep disorders, such as obstructive sleep apnea (OSA) and obesity-related sleep disturbances.

## Methods

A literature search was initiated on September 13, 2022, using five medical databases: Scopus, Cochrane Library, Google Scholar, PubMed, and WebMD. This systematic search process was reiterated on October 20, 2022, January 15, 2023, and April 1, 2023, employing specific search terms as free text, including (plant-rich diet OR plant-rich diet) and (fiber OR fiber-rich diet OR dietary fiber) and (sleep quality OR sleep characteristics or sleep) and (obstructive sleep apnea OR daytime sleepiness). From the 267 discerned studies, 68 (25.5%) met the following inclusion criteria: alignment with at least two of the prescribed keywords, garnering 10 or more citations, and a publication date after 1990.

For each of these selected studies, the following data was extracted from each article: authorship, year of publication, methodologic approach, participant count, study conclusions, and quantity of citations. The 68 included articles were then systematically classified into seven distinct thematic categories: (1) high-fat diet and sleep (8 studies); (2) fiber-rich diet and sleep (12 studies); (3) gut microbiome and sleep (6 studies); (4) tryptophan/melatonin and sleep (8 studies); (5) inflammation and sleep (3 studies); (6) polyphenols and sleep (4 studies); and (7) diet, weight, and OSA (27 studies) (Supplementary Table S1). Simultaneously, another distinct cohort of 193 articles focusing on the ramifications of PRDs on chronic conditions and mortality was identified. Of these, 56 (29.0%) were selected and further organized into five subcategories: (1) diabetes mellitus and PRD; (2) cardiovascular diseases and PRD; (3) all mortality decrease and PRD; (4) obesity, weight changes, and PRD; (5) cancer and PRD (Supplementary Table S2).

Subsequent to this classification, a reevaluation and analysis of each article across the aforementioned categories and subcategories was conducted by our research team. The major findings from the articles in Supplementary Table S1 were then summarized in the discussion section below. Articles listed in Supplementary Table S1 were only discussed if the research was conducted in human subjects and if the research investigated the effects of dietary intake on sleep, rather than the effects of sleep on dietary intake. Studies involving micronutrients, rather than the use of whole foods, were also excluded, as were studies on dietary manipulations other than increasing the intake of plants. The articles in Supplementary Table S2 served as additional background reading, some of which is referenced in the introduction section.

## Discussion

### High-fat diets and sleep

A notable aspect of plant-rich diets is their characteristic low-fat profile. Several research studies have suggested a connection between a diet rich in saturated fats and the onset of drowsiness.

One investigation involving 459 participants (4) explored the idea that excessive fat consumption might hinder lipid synthesis and delay eIF2a phosphorylation, resulting in reduced signals for sleep, ultimately leading to shorter nighttime sleep and increased daytime sleepiness. The precise mechanism behind this phenomenon remains inadequately understood, but it is theorized that elevated fat intake could impede lipid synthesis and eIF2a phosphorylation, consequently diminishing the sleep signal.

In another study (5), the impact of high-fat meals on postprandial sleepiness was examined, with a focus on the elevation of CCK concentration. High-fat meals trigger the release of cholecystokinin from the duodenum, which in turn activates specific receptors on the vagal nerve, leading to an increased firing rate of its afferent nerves (6). Consequently, this activation of the vagal nerve contributes to reduced alertness (7).

A crossover study (8) also revealed a negative correlation between dietary fat intake (as well as carbohydrates) and the proportion of time spent in N3 sleep, indicating that higher fat consumption may affect the quality of sleep.

### Fiber-rich diets and sleep

Research from countries around the world has indicated a potentially beneficial role of dietary fiber in sleep quality. One randomized crossover interventional study demonstrated that dietary patterns with higher fiber content were associated with improved sleep quality, with a significant positive correlation between fiber intake and time spent in slow-wave sleep. Moreover, fiber consumption was linked to a shorter time in stage N1 sleep, a light stage of sleep (9). A population-based study also found that a higher intake of vegetables was associated with improved sleep quality (10).

Several established diet patterns, such as the Mediterranean, Dietary Approaches to Stop Hypertension (DASH), Mediterranean-DASH Intervention for Neurodegenerative Delay (MIND), and vegetarian diets, consistently incorporate high dietary fiber through fruits, vegetables, whole grains, and legumes. These dietary habits have been the subject of numerous studies, all of which have demonstrated the positive impact of fiber-rich foods on sleep characteristics (11–13).

A study investigating the relationship between sleep patterns, body mass index, and adherence to the Mediterranean diet in young adolescents found a significant linear association between higher diet adherence and lower daytime sleepiness (14). Higher fruit and vegetable intake were also associated with increased sleep duration. Similarly, a cross-sectional study on adolescent girls showed an inverse correlation between adherence to the DASH diet and daytime sleepiness (15). Participants with significantly higher intakes of fruits, vegetables, and nuts, along with lower consumption of refined grains, red and processed meat, sugar-sweetened beverages, and sweets, had a lower tendency toward daytime sleepiness. Moreover, a prospective cohort study evaluating the effect of the Mediterranean diet on sleep parameters in US women reported that fruit and vegetable consumption predicted a lower Pittsburgh Sleep Quality Index (PSQI) score (improved sleep quality), better sleep efficiency, and fewer sleep disruptions (16). A study involving adults in the UK indicated that individuals who typically slept for 7–8 h per day, known as reference sleepers, had the highest levels of fruit and vegetable intake compared to those who slept for shorter or longer durations (12). Recent studies have also shown that eating more fruits and vegetables is linked to better sleep quality and quicker time to fall asleep in men (17) and improved sleep issues related to insomnia in young women who previously consumed less than three servings of fruits and vegetables per day (18).

A cohort study of 495 women found that a lower quality diet containing fewer whole grain foods was associated with poor sleep quality (19). Another cross-sectional study of 410 female students in Iran found that women sleeping <6 h per night consumed significantly fewer high fiber foods than women who obtained more sleep (20). A cross-sectional study of 2050 Japanese workers found that high intakes of foods high in fiber was associated with a reduced prevalence of difficulty falling asleep (21). Data from the NHANES database looking at dietary quality and sleep duration in US women within 5 years of childbirth found that women with long sleep (>9 h) had worse dietary quality than those who slept <9 h. Overall dietary quality in this population of women was very low (total score below 50% of the maximum score) (22).

Some possible mechanisms by which increased dietary fiber might improve sleep quality include stabilizing blood sugar levels and improving the gut microbiome.

### Gut microbiome and sleep

Studies have established a robust association between dietary patterns and the composition of the gut microbiome (23, 24). Notably, this research highlights the favorable impact of plant-rich diets, particularly those inspired by Mediterranean and vegetarian dietary patterns, on the gut’s bacterial flora (25–27). A study involving 106 male patients with coronary heart disease demonstrated that long-term adherence to healthy eating habits, including low-fat and Mediterranean diets, reduced gut dysbiosis among study participants with metabolic syndrome (28). Additionally, a recent review of randomized controlled trials emphasized the potential of dietary fiber to influence the composition of the gut bacterial flora through several mechanisms, such as modifying the Prevotella/Bacteroides ratio, expanding the population of bacteria that produce short-chain fatty acids, and promoting overall bacterial growth (29).

While these studies underscore the link between diet and gut microbiome composition, no research to date has explicitly explored whether modifying the gut microbiome through dietary means can directly enhance sleep quality. Nevertheless, several studies have indirectly indicated a relationship between improved sleep and indicators of a balanced gut flora (30). Moreover, some investigations have documented changes in the gut microbiome in response to sleep disruptions and circadian rhythm disturbances (31–35).

A double-blind, randomized, placebo-controlled trial examined the effects of probiotics, particularly *Lactobacillus casei* Shirota–fermented milk, on various sleep parameters (36). This trial revealed a significant *Lactobacillus casei* Shirota treatment effect of reduced drowsiness upon awakening and increased subjective sleep duration.

Additionally, there is some data on the use of clarithromycin as a treatment for idiopathic hypersomnia and its potential mechanisms of action related to the gut microbiome (37). The study suggested that clarithromycin’s anti-inflammatory properties and its ability to modulate the gut microbiome could contribute to its therapeutic effects.

Future dietary interventions within controlled trials are essential to determine whether there is a causal relationship between changes in sleep parameters and modifications of the gut microbiome induced by dietary strategies, including PRDs. Such investigations hold the potential to shed further light on the intricate interplay between diet, the gut microbiome, and sleep quality.

### Plants as a source of melatonin and its precursors

Melatonin is a hormone critical for regulating sleep–wake cycles, and its precursors, tryptophan, and serotonin, are essential for healthy sleep. Notably, plants are a primary dietary source of melatonin, with higher average concentrations found in plant cells compared to animal cells (38). A meta-analysis of 17 studies on the effects of exogenous melatonin supplementation revealed significant improvements in total sleep duration, sleep onset latency, and sleep efficiency (39). It is postulated that consuming plant-rich foods enhances melatonin production but more research is needed.

### Effect of plants on systemic inflammation and sleep

Chronic inflammation has been linked to sleep disturbances. A meta-analysis of 72 studies of over 50,000 individuals found that sleep disturbance and extremely long sleep duration were associated with increased levels of proinflammatory cytokines, CRP and IL-6 (40). A cross-sectional study of almost 2000 Italian adults found individuals with a higher dietary inflammatory index were less likely to have adequate sleep quality (41). Diets rich in anti-inflammatory components, such as vitamin E in nuts and olive oil, folate in green leafy vegetables, omega-3 fatty acids in fish and walnuts, and flavonoids in berries, may therefore benefit individuals with sleep disturbances, including daytime sleepiness (42).

### Isoflavones and sleep

Isoflavones, a subgroup of phytoestrogens found in legumes like chickpeas, soybeans, fava beans, peanuts, pistachios, and various nuts and fruits, have mild estrogenic effects that resemble human estrogen. Since estrogen plays a role in regulating sleep duration and quality, researchers have hypothesized that isoflavones might similarly impact sleep. A cross-sectional study involving a Japanese population discovered a positive correlation between higher daily isoflavone consumption and improved sleep duration and quality (43). Furthermore, a randomized, controlled, double-blind study conducted on postmenopausal women experiencing insomnia demonstrated a significant enhancement in sleep efficiency, measured by polysomnography, among those in the isoflavone group compared to the placebo group (44). A 2017 study of a Chinese population over a five-year period revealed that individuals in the highest quartile of isoflavone intake were less likely to experience overly long sleep durations (45). Additionally, persistently high isoflavone intake over 5 years was substantially associated with a reduced risk of daytime sleepiness in women.

### Diet, weight, obstructive sleep apnea, and sleep quality

Obstructive Sleep Apnea (OSA) is characterized by sleep fragmentation and daytime drowsiness. While weight loss has been shown to improve OSA, dietary factors independent of weight loss may play a crucial role (46). An extensive study that comprised three prospective cohorts of 8,856 patients with OSA (47) revealed an association between diet quality (determined by Alternative Healthy Eating Index 2010 and Empirical Dietary Inflammatory Pattern scores) and risk of OSA. Specifically, after carefully removing all relevant confounders, the authors established that worse diet quality and higher dietary inflammatory potential were associated with increased OSA risk.

In contrast, transitioning to whole-food plant-rich diets has demonstrated promising results in reducing daytime sleepiness among patients with. In a recent study, Patel et al. (48) studied a cohort of 14 patients with OSA with complaints of daytime sleepiness in an outpatient sleep clinic. Patients changed their diet from a Western diet to a whole-food plant-rich diet for 3 weeks. Their mean Epworth Sleepiness Scale score reduced significantly by an average of 3.8.

Independent of OSA, several cross-sectional studies have highlighted the connection between obesity, sleep disturbances, and drowsiness (49). PRDs are effective for weight loss and maintenance, and studies have shown that a vegan diet leads to lower body weight (50) and less weight gain (51) compared to other PRDs (i.e., omnivorous, semi-vegetarian, pescovegetarian, and vegetarian diets).

Dietary interventions emphasizing micronutrient-dense, plant-rich diets have shown promise in enhancing sleep quality in multiple studies. A 9-week trial of one such dietary intervention in a cohort of employees found that Pittsburgh Sleep Quality Index (PSQI) scores improved from a median score of 8 to 4, consistent with an improvement in the participants’ sleep quality (52). In another study conducted at a university setting, 35 individuals switched their diet to a micronutrient-dense, plant-rich diet for 6 weeks. The PSQI scores again improved from a mean score of 6.6–3.4 (53).

### Limitations and future directions

While epidemiologic studies suggest a positive association between plant-rich diets and sleep, further interventional research is crucial to establish causality and provide practical recommendations for clinicians. Even in randomized trials, transitioning to a PRD is often associated with a reduction in body weight so it can be hard to sort out whether some of the benefits seen might be due to weight loss alone versus direct benefits of plants. Future trials that compare caloric restriction alone to a plant rich diet might be better able to distinguish between these effects.

## Conclusion

In summary, plant-rich diets, characterized by their high fiber content, melatonin precursors, isoflavones and positive effects on the gut microbiome, hold promise in improving sleep quality and addressing sleep-related disorders, including OSA and obesity-associated sleep disturbances. As we strive for a more comprehensive understanding of the relationship between plant-rich diets and sleep, future research will play a pivotal role in advancing this field. Clinicians should consider advocating for the incorporation of minimally processed, fiber-rich plant foods in holistic approaches to managing sleep problems.

## Author contributions

AP and JC were involved in conception of this review. AP conducted the literature search. All authors were involved in the writing and final approval of this article.
