# Supplementary material for: The impact of plant-rich diets on sleep: a mini-review
Source: Front Nutr. 2024 Feb 6;11:1239580. doi: 10.3389/fnut.2024.1239580 (PMC10876799; doi:10.3389/fnut.2024.1239580)
Supplement: Supplementary file 1 [file Table_1.docx]

Supplemental Table 1.

| Document title | Authors | Year | Source | Results, conclusions |
| --- | --- | --- | --- | --- |
| Relationships among dietary nutrients and subjective sleep, objective sleep, and napping in women. | Grandner, M.A., Kripke, D.F., Naidoo, N., Langer, R.D. | 2010 | [Sleep Medicine](https://www.sciencedirect.com/science/article/pii/S138994570900344X?via%3Dihub)  [11(2), pp. 180-184](https://www.sciencedirect.com/science/article/pii/S138994570900344X?via%3Dihub) | Actigraphic total sleep time was negatively associated with intake of fats. Subjective napping, which may be a proxy for subjective sleepiness, was significantly related to fat intake as well as intake of meat. |
| Sleep symptoms associated with intake of specific dietary nutrients | Grandner, M.A., Jackson, N., Gerstner, J.R., Knutson, K.L. | 2014 | [Journal of Sleep Research](https://onlinelibrary.wiley.com/doi/10.1111/jsr.12084)  [23(1), pp. 22-34](https://onlinelibrary.wiley.com/doi/10.1111/jsr.12084) | Daytime sleepiness was associated with increased caloric intake in adjusted analyses. It was also associated with higher likelihood of less food reported compared to usual diet in unadjusted analyses only, and being on a low fat/cholesterol diet in both unadjusted and adjusted analyses. Finally, the nutrients that were associated significantly with greater daytime sleepiness were, in order, more moisture, more theobromine, less potassium and less plain water.Difficulty maintaining sleep was associated with fewer foods in the diet and, along with daytime sleepiness, was associated with being on a special diet. Being on a low fat/cholesterol diet was associated with less non-restorative sleep and daytime sleepiness. |
| Alterations in sleep architecture in response to experimental sleep curtailment are associated with signs of positive energy balance | Shechter, A., O'Keeffe, M., Roberts, A.L., (...), RoyChoudhury, A., St-Onge, M.-P. | 2012 | [American Journal of Physiology - Regulatory Integrative and Comparative Physiology](https://www.scopus.com/record/display.uri?eid=2-s2.0-84868371030&origin=resultslist&sort=plf-f&src=s&st1=Alterations+in+sleep+architecture+in+response+to+experimental+sleep+curtailment+are+associated+with+signs+of+positive+energy+balance&sid=79a24df633a8bf6bc90cf475123ca8fa&sot=b&sdt=b&sl=147&s=TITLE-ABS-KEY%28Alterations+in+sleep+architecture+in+response+to+experimental+sleep+curtailment+are+associated+with+signs+of+positive+energy+balance%29&relpos=0&citeCnt=66&searchTerm=)  [303(9), pp. R883-R889](https://www.scopus.com/record/display.uri?eid=2-s2.0-84868371030&origin=resultslist&sort=plf-f&src=s&st1=Alterations+in+sleep+architecture+in+response+to+experimental+sleep+curtailment+are+associated+with+signs+of+positive+energy+balance&sid=79a24df633a8bf6bc90cf475123ca8fa&sot=b&sdt=b&sl=147&s=TITLE-ABS-KEY%28Alterations+in+sleep+architecture+in+response+to+experimental+sleep+curtailment+are+associated+with+signs+of+positive+energy+balance%29&relpos=0&citeCnt=66&searchTerm=) | Reduced N3 sleep has been found to be associated with poor diet intake. In the study, percentage of time spent in N3 sleep was inversely associated with fat and carbohydrate intake. This study demonstrates that changes in sleep architecture are associated with markers of positive energy balance and indicate a means by which exposure to short sleep duration and/or an altered sleep architecture profile may lead to excess weight gain over time. |
| Increased food intake by insufficient sleep in humans: Are we jumping the gun on the hormonal explanation? | Chaput, J.-P., St-Onge, M.-P. | 2014 | [Frontiers in Endocrinology, 5 (JUL), art. no. Article 116](https://www.scopus.com/record/display.uri?eid=2-s2.0-84905382904&origin=reflist&sort=r-f&src=s&st1=sleep+and+diet%3a+mounting&nlo=&nlr=&nls=&sid=3b72eb5df3cca5f1d54b130d6dd7195e&sot=b&sdt=b&sl=39&s=TITLE-ABS-KEY%28sleep+and+diet%3a+mounting%29) | Results: No differences in sleep patterns were observed in either gender, except in the percentage of stage 1 sleep, which was greater in men. Different correlations were observed between sleep and dietary variables according to gender. The correlation between dietary and sleep variables in men indicated a negative relationship between nocturnal fat intake and the sleep latency, including REM sleep. The percentage of nocturnal fat intake correlated with sleep efficiency, sleep latency, REM latency, stage 2 sleep, REM sleep, and wake after sleep onset (WASO) in women. The percentage of nocturnal caloric intake correlated with sleep latency and efficiency in women. |
| Short sleep duration and dietary intake: epidemiologic evidence, mechanisms, and health implications | Dashti, H.S., Scheer, F.A.J.L., Jacques, P.F., Lamon-Fava, S.,  Ordovás, J.M. | 2015 | [Advances in Nutrition, 6(6), pp. 648–65](https://linkinghub.elsevier.com/retrieve/pii/S2161831323001138)  [9](https://linkinghub.elsevier.com/retrieve/pii/S2161831323001138) | The studies have observed consistent associations between short sleep duration and higher total energy intake and higher total fat intake, and limited evidence for lower fruit intake, and lower quality diets. Evidence also suggests that short sleepers may have irregular eating behavior deviating from the traditional 3 meals/d to fewer main meals and more frequent, smaller, energy-dense, and highly palatable snacks at night. |
| Suppression of food intake by apolipoprotein A-IV is mediated through the central nervous system in rats. | Fujimoto, K., Fukagawa, K., Sakata, T. & Tso, P. | 1993 | [Journal of Clinical Investigation](https://pubmed.ncbi.nlm.nih.gov/8473522/)  [91(4), pp. 1830-1833](https://pubmed.ncbi.nlm.nih.gov/8473522/) | apoprotein A-IV secreted from the intestine after a fatty meal may act on receptors in the central nervous system to alter eating behaviour, but so far no change in sedation has been observed |
| Influences of fat and carbohydrate on postprandial sleepiness, mood, and hormones. | Wells AS, Read NW, Uvnas-Moberg K, Alster P. | 1997 | *Physiol Behav*. 1997;61(5):679-686. doi:10.1016/s0031-9384(96)00519-7 | Subjects tended to feel more sleepy and less awake 2-3 h after the high-fat-low-CHO meal, and ratings of fatigue were significantly greater 3 h after the high-fat-low-CHO meal than after the low-fat-high-CHO meal. The results of the present study are consistent with the hypothesis that there is an association between the lassitude experienced after a meal and the release of CCK. |
| Relationship between food intake and sleep pattern in healthy individuals | Crispim CA, Zimberg IZ, dos Reis BG, Diniz RM, Tufik S, de Mello MT. | 2011 | J Clin Sleep Med. 7(6), pp. 659-64. | Food intake during or near the nocturnal period was correlated with negative effects on the sleep quality of healthy individuals |
| Relationships among dietary nutrients and subjective sleep, objective sleep, and napping in women | Grandner MA, Kripke DF, Naidoo N, Langer RD. | 2010 | *Sleep Med*. 2010;11(2):180-184. doi:10.1016/j.sleep.2009.07.014 | Participants were 459 post-menopausal women enrolled in the Women's Health Initiative. Objective sleep was estimated using one week of actigraphy. Subjective sleep was prospectively estimated with a daily sleep diary. he most significant correlations were with subjective napping, including (from strongest to weakest): total fat, calories, saturated fat, monounsaturated fat, trans fat, water, proline, serine, tyrosine, phenylalanine, valine, cholesterol, leucine, glutamic acid, ash, isoleucine, histidine, sodium, tryptophan, protein, threonine, cystine, methionine, phosphorous, polyunsaturated fat, animal protein, aspartic acid, arginine, lysine, alanine, caffeine, riboflavin, gamma-tocopherol, glycine, retinol, delta-tocopherol, Vitamin D, and selenium. Actigraphic nocturnal sleep duration was negatively associated with total fat, monounsaturated fat, trans fat, saturated fat, polyunsaturated fat, calories, gamma-tocopherol, cholesterol, and alpha-tocopherol-eq. |

| Document title | Authors | Year | Source | Results, conclusions |
| --- | --- | --- | --- | --- |
| A Mediterranean Dietary Pattern Predicts Better Sleep Quality in US Women from the American Heart Association Go Red for Women Strategically Focused Research Network | Zuraikat, F.M., Makarem, N., St-Onge, M.-P., (...), Akkapeddi, A., Aggarwal, B. | 2020 | [Nutrients](https://www.mdpi.com/2072-6643/12/9/2830)  [12(9),2830, pp. 1-13](https://www.mdpi.com/2072-6643/12/9/2830) | Higher baseline aMed scores were associated with lower PSQI scores (β = −0.30 ± 0.10, p < 0.01), indicative of better sleep quality, higher sleep efficiency (β = 1.20 ± 0.35, p < 0.001), and fewer sleep disturbances (β = −0.30 ± 0.12, p = 0.01) at 1-y. Fruit and vegetable consumption also predicted lower PSQI scores, higher sleep efficiency and fewer sleep disturbances (all p < 0.05). Higher legume intake predicted better sleep efficiency (β = 1.36 ± 0.55, p = 0.01). These findings suggest that adherence to a Mediterranean diet pattern should be evaluated as a strategy to promote sleep quality in US women |
| Fiber and saturated fat are associated with sleep arousals and slow wave sleep | St-Onge MP, Roberts A, Shechter A, Choudhury AR | 2016 | [Journal of Clinical Sleep Medicine](https://jcsm.aasm.org/doi/10.5664/jcsm.5384)  [12(1), pp. 19-24](https://jcsm.aasm.org/doi/10.5664/jcsm.5384) | Low fiber and high saturated fat and sugar intake is associated with lighter, less restorative sleep with more arousals. Diet could be useful in the management of sleep disorders but this needs to be tested. |
| Sleep deprivation is associated with lower diet quality indices and higher rate of general and central obesity among young female students in Iran. | Fahimeh Haghighatdoost 1, Golgis Karimi, Ahmad Esmaillzadeh, Leila Azadbakht | 2012 | [Nutrition. 2012;28(11-12):1146–1150.](https://pubmed.ncbi.nlm.nih.gov/22951155/) | Results: Subjects who slept less than 6 h/d were more likely to be overweight and obese and also abdominally obese. They also consumed more dietary energyand carbohydrates but a lower amount of fiber, fruits, whole grains, and beans. All diet quality indices were significantly lower among short sleepers, apart from dietary energy density, which did not differ significantly . Conclusion: Our findings confirm the association of short sleep duration and obesity in young female youths. It might be derived from lower diet quality among short sleepers more than longer sleepers. |
| Sleep quality and duration is related with diet and obesity in young adolescent living in Sicily, Southern Italy | Ferranti, R., Marventano, S., Castellano, S., (...), Matalone, M., Mistretta, A. | 2016 | [Sleep Science 9(2), pp. 117-122](https://pubmed.ncbi.nlm.nih.gov/27656277/) | Sleep disturbance increase morning cortisol levels, reduce insulin sensitivity and growth hormone secretion [37], [38], supporting the associations between poor sleep quality and obesity, as found in other studies [39], [40]. In conclusion, less sleep and poor sleep were associated with a lower adherence to Mediterranean Diet, an increase in unhealthy eating behaviors and overweigh/obese status. Moreover, adolescents with late bedtime had a higher intake of extra foods (energy-dense, nutrient-poor foods) while adolescent with an early bedtime consume more fruit and vegetables. Despite its association with BMI in adolescents is still unclear [55], a high adherence to a healthy dietary pattern, such as the Mediterranean diet, may be associated with a whole healthier lifestyle, which include also better sleep quality. |
| Low intake of vegetables, high intake of confectionary, and unhealthy eating habits are associated with poor sleep quality among middle-aged female Japanese workers | Katagiri, R., Asakura, K., Kobayashi, S., Suga, H., Sasaki, S. | 2014 | [Journal of Occupational Health](https://onlinelibrary-wiley-com.rossuniversity.idm.oclc.org/doi/abs/10.1539/joh.14-0051-OA)  [56(5), pp. 359-368](https://onlinelibrary-wiley-com.rossuniversity.idm.oclc.org/doi/abs/10.1539/joh.14-0051-OA) | Poor sleep quality was associated with low intake of vegetables (p for trend 0.002) and fish (p for trend 0.04) and high intake of confectionary (p for trend 0.004) and noodles (p for trend 0.03) after adjustment for potential confounding factors (age, body mass index, physical activity, depression score, employment status, alcohol intake and smoking status). Poor sleep quality was also significantly and positively associated with consumption of energy drinks and sugar-sweetened beverages, skipping breakfast, and eating irregularly. In addition, poor sleep quality was significantly associated with high carbohydrate intake (p for trend 0.03). |
| Dietary patterns and sleep symptoms in Japanese workers: The Furukawa Nutrition and Health Study | Kurotani, K., Kochi, T., Nanri, A., (...), Kabe, I., Mizoue, T. | 2015 | [Sleep Medicine](https://www-sciencedirect-com.rossuniversity.idm.oclc.org/science/article/abs/pii/S1389945714004869?via%3Dihub)  [16(2), pp. 298-304](https://www-sciencedirect-com.rossuniversity.idm.oclc.org/science/article/abs/pii/S1389945714004869?via%3Dihub) | We identified three major dietary patterns. A healthy pattern, characterized by a high intake of vegetables, mushrooms, potatoes, seaweeds, soy products, and eggs, was associated with a decreased prevalence of difficulty initiating sleep once or more a week (P for trend = 0.03); the multivariate adjusted odds ratio in the highest quartile of this score compared with the lowest was 0.75 (95% CI: 0.57–0.99). This association persisted after the exclusion of individuals with severe depressive symptoms. However, there was no significant association with difficulty initiating sleep at least three times a week. Conclusions: Our findings suggest that a healthy dietary pattern may be associated with difficulty initiating sleep at least once a week. |
| Changes in fruit and vegetable consumption in relation to changes in sleep characteristics over a 3-month period among young adults | Jansen, E.C., She, R., Rukstalis, M., Alexander, G.L. | 2021 | [Sleep Health](https://www.sciencedirect.com/science/article/pii/S2352721821000139?via%3Dihub)  [7(3), pp. 345-352](https://www.sciencedirect.com/science/article/pii/S2352721821000139?via%3Dihub) | Results: Average age ± SD was 26 ± 2.8 years (71% women). At 3-month follow-up, participants on average increased FV intake by 1.2 ± 1.4 servings. Women who increased FV intake by 3+ servings showed improvements in insomnia symptoms (2-fold higher odds of improvement; 95% CI 1.1 to 3.6), sleep quality (0.2-point higher sleep quality score; 95% CI -0.01, 0.3), and time to fall asleep (4.2 minutes; 95% CI -8, 0) compared to women who did not change or decreased their FV intake. Associations were not as apparent among men. Conclusion: Young women with low consumption of FV (fruit and vegetable) may experience improvements in insomnia-related sleep difficulties by increasing their consumption of FV. |
| Sleep Duration and Quality in Relation to Fruit and Vegetable Intake of US Young Adults: a Secondary Analysis | Jansen, E.C., She, R., Rukstalis, M.M., Alexander, G.L. | 2021 | [International Journal of Behavioral Medicine](https://link.springer.com/article/10.1007/s12529-020-09853-0)  [28(2), pp. 177-188](https://link.springer.com/article/10.1007/s12529-020-09853-0) | After accounting for confounders, men with better sleep quality and shorter time to fall asleep had higher intakes of FV (1.12 serving/day difference in highest versus lowest quality [95% CI 0.48, 1.75] and a 0.52 serving/day higher intake difference for shortest versus longest fall asleep time [95% CI 0.90, 0.15], respectively).Conclusion: Sleep was highly prevalent in a diverse sample of community-based young adults and may contribute to lower FV intake among men. These associations highlight young adulthood as an important period for promoting healthy sleep habits. |
| Adherence to a dietary approach to stop hypertension (DASH)-style in relation to daytime sleepiness. | Pahlavani N, Khayyatzadeh SS, Banazadeh V, Bagherniya M, Tayefi M, Eslami S, et al. | 2020 | [Nature and Science of Sleep](https://www.dovepress.com/getfile.php?fileID=58689)  [12, pp. 325-332](https://www.dovepress.com/getfile.php?fileID=58689) | As may be expected, participants with the greatest adherence to the DASH diet had significantly higher intakes of fruits, vegetables, low-fat dairy products, fish and nuts, and lower consumption of refined grains, red and processed meat, sugar-sweetened beverages and sweets. ThThese findings were remained signifi-cant after adjustment for confounding variables (β= −0.08 P=0.04). Conclusion: There is an inverse correlation between adherence to DASH diet and daytime sleepiness score. Further studies, particularly longitudinal studies, are required to determine whether dietary intervention may improve daytime sleepiness. |
| Adherence to the MIND dietary pattern and sleep quality, sleep related outcomes and mental health in male adults: a cross-sectional study | Rostami, H., Parastouei, K., Samadi, M., Taghdir, M., Eskandari, E. | 2022 | [BMC Psychiatry](https://pubmed.ncbi.nlm.nih.gov/35248010/)  [22(1),167](https://pubmed.ncbi.nlm.nih.gov/35248010/) | Participants in the highest tertile of MIND diet had a 42% lower odds of daytime sleepiness in the crude and multivariable-adjusted model (P-trend < 0.05). While no significant associations were found between adherence to the MIND diet and stress, anxiety and depression, greater adherence to the MIND diet were associated with lower odds of poor sleep quality and sleep-related outcomes. |
| Sleep duration and diet quality among women within 5 years of childbirth in the United States: a cross-sectional study. | Xiao RS, Simas TAM, Pagoto SL, Person SD, Rosal MC, Waring ME. | 2016 | [Maternal and Child Health Journal](https://link-springer-com.rossuniversity.idm.oclc.org/article/10.1007/s10995-016-1991-3)  [20(9), pp. 1869-1877](https://link-springer-com.rossuniversity.idm.oclc.org/article/10.1007/s10995-016-1991-3) | Thirty-four percent of women reported short, 57.1 % adequate, and 8.6 % long sleep duration. The average diet quality total score was 47.4 out of 100. Short sleep duration was not associated with diet quality. Long sleep duration was associated with lower quality diet, lower consumption of total fruit ), whole fruit , and total protein), and higher consumption of empty calories . Conclusions for practice Future studies should examine the longitudinal association between sleep duration and diet quality among women following childbirth and whether interventions to improve sleep can enhance diet quality. |
| Vitamin C and fibre consumption from fruits and vegetables improves oxidative stress markers in healthy young adults | Hermsdorff, H.H.M., Barbosa, K.B.F., Volp, A.C.P., (...), Zulet, M.A., Martínez, J.A. | 2012 | [British Journal of Nutrition](https://www.cambridge.org/core/journals/british-journal-of-nutrition/article/vitamin-c-and-fibre-consumption-from-fruits-and-vegetables-improves-oxidative-stress-markers-in-healthy-young-adults/29CF37B94B4BEB6D1F8EFEA1126A25E8)  [107, pp. 1119-1127](https://www.cambridge.org/core/journals/british-journal-of-nutrition/article/vitamin-c-and-fibre-consumption-from-fruits-and-vegetables-improves-oxidative-stress-markers-in-healthy-young-adults/29CF37B94B4BEB6D1F8EFEA1126A25E8) | Those subjects in the highest tertile (T) of FV consumption ( ≥ 705 g/d) had statistically lower oxidised LDL (ox-LDL) concentrations as well as higher plasma total antioxidant capacity (TAC) and glutathione peroxidase (GPx) activity (P for trend < 0·05), after adjusting for sex, age, energy intake, physical activity, smoking, BMI, vitamin supplement use and other confounding factors. Moreover, plasma ox-LDL concentrations showed a decreasing trend and TAC an increasing trend across tertiles of fibre (T3: ≥ 14 g/d) and vitamin C (T3: ≥ 150 mg/d) from FV intake, while GPx activity was positively associated with vitamin C intake (P for trend < 0·05). In conclusion, greater FV consumption was independently associated with reduced ox-LDL as well as increased TAC and GPx activity in healthy young adults, with dietary fibre and vitamin C from FV clearly being implicated in this beneficial relationship. |
| Measures of Poor Sleep Quality Are  Associated With Higher Energy Intake and Poor Diet Quality in a Diverse Sample of Women From the Go Red for Women Strategically Focused Research Network. | Zuraikat, F.M.; Makarem, N.; Liao, M.; St-Onge, M.-P.; Aggarwal, B. | 2020 | [Journal of the American Heart Association](https://www.ahajournals.org/doi/10.1161/JAHA.119.014587)  [9(4),e0145](https://www.ahajournals.org/doi/10.1161/JAHA.119.014587)  [87](https://www.ahajournals.org/doi/10.1161/JAHA.119.014587) | Results showed that higher PSQI scores, indicative of poorer sleep quality, were associated with lower unsaturated fat intake (β=−0.14, P<0.05) and higher food weight (β=14.9, P=0.02) and added sugars consumed (β=0.44, P=0.04). Conclusions: Poor sleep quality was associated with greater food intake and lower‐quality diet, which can increase cardiovascular disease risk. Future studies should test whether promoting sleep quality could augment efforts to improve cardiometabolic health in women. |
| Plant-Based Diets: Reducing Cardiovascular Risk by Improving Sleep Quality? | St-Onge, M.-P., Crawford, A., Aggarwal, B. | 2018 | [Current Sleep Medicine Reports](https://link.springer.com/article/10.1007/s40675-018-0103-x)  [4(1), pp. 74-78](https://link.springer.com/article/10.1007/s40675-018-0103-x) | Findings: Short sleep duration and poor sleep quality have been shown to negatively affect individual dietary habits, through enhanced appetite and increased overall caloric intake, as well as lower diet quality. Emerging data also indicate a novel bi-directional association by which dietary choices may also influence sleep duration and quality, but little is known about dietary patterns and their influence on sleep. Epidemiological studies report associations between Mediterranean diet eating patterns and sleep quality, suggesting a benefit of plant-rich diet consumption on sleep. The high isoflavone and tryptophan content of these diets may be a mechanism by which plant foods may enhance sleep quality. Summary: Plant-based diets may provide additional benefits to health via their potential effects on sleep quality. Research is needed to establish a causal relation between a plant-rich dietary pattern and sleep health. |
| The relationship between sleep duration and fruit/vegetable intakes in UK adults: a cross-sectional study from the National Diet and Nutrition Survey | Noorwali EA, Cade JE, Burley VJ, et al | 2018 | BMJ Open | Both short and long sleepers, compared to reference sleepers, consumed fewer fruits and vegetables. |
| Actigraphic sleep measures and diet quality in the Hispanic Community Health Study/Study of Latinos Sueño ancillary study. | Mossavar-Rahmani Y, Weng J, Wang R, Shaw PA, Jung M, Sotres-Alvarez D, Castañeda SF, Gallo LC, Gellman MD, Qi Q, Ramos AR, Reid KJ, Van Horn L, Patel SR. | 2017 | J Sleep Res. | “Both longer sleep duration and higher sleep efficiency were significantly associated with better diet quality among US Hispanic/Latino adults.” |

| Document title | Authors | Year | Source | Results, conclusions |
| --- | --- | --- | --- | --- |
| Reciprocal roles of sleep and diet in cardiovascular health: a review of recent evidence and a potential mechanism | St-Onge MP, Zuraikat FM. | 2019 | [Current Atherosclerosis Reports, 21(3), 11](https://link.springer.com/article/10.1007/s11883-019-0772-z) | There is growing evidence of a bi-directional relationship between sleep and the diet, which could act in concert to influence CVD risk. Diets such as the Mediterranean diet, comprised of high intakes of fruits, vegetables, and other plant-based foods, may promote healthy sleep and beneficial gut microflora. The gut microbiome may then underlie the relation between diet, sleep, and CVD risk. |
| Long-term dietary intervention reveals resilience of the gut microbiota despite changes in diet and weight | Fragiadakis, G.K., Wastyk, H.C., Robinson, J.L., (...), Sonnenburg, J.L., Gardner, C.D. | 2020 | [American Journal of Clinical Nutrition](https://academic.oup.com/ajcn/article/111/6/1127/5809430)  [111(6), pp. 1127-1136](https://academic.oup.com/ajcn/article/111/6/1127/5809430) | While baseline microbiota composition was not predictive of weight loss, each diet resulted in substantial changes in the microbiota 3-mo after the start of the intervention; some of these changes were diet specific (14 taxonomic changes specific to the healthy low-carbohydrate diet, 12 taxonomic changes specific to the healthy low-fat diet) and others tracked with weight loss (7 taxonomic changes in both diets). Conclusions: these results suggest a resilience to perturbation of the microbiota's starting profile. When considering the established contribution of obesity-associated microbiotas to weight gain in animal models, microbiota resilience may need to be overcome for long-term alterations to human physiology. |
| Influence of diet on the gut microbiome and implications for human health. | Singh RK, Chang H-W, Yan D, Lee KM, Ucmak D, Wong K, et al | 2017 | [Journal of Translational Medicine](https://www.scopus.com/record/display.uri?eid=2-s2.0-85018493988&origin=resultslist&sort=r-f&src=s&st1=Influence+of+diet+on+the+gut+microbiome+and+implications+for+human+health.&nlo=&nlr=&nls=&sid=eeeb48fe6ce45f0e9beabcc134c4e060&sot=b&sdt=b&sl=89&s=TITLE-ABS-KEY%28Influence+of+diet+on+the+gut+microbiome+and+implications+for+human+health.%29&relpos=0&citeCnt=1145&searchTerm=)  [15(1),73](https://www.scopus.com/record/display.uri?eid=2-s2.0-85018493988&origin=resultslist&sort=r-f&src=s&st1=Influence+of+diet+on+the+gut+microbiome+and+implications+for+human+health.&nlo=&nlr=&nls=&sid=eeeb48fe6ce45f0e9beabcc134c4e060&sot=b&sdt=b&sl=89&s=TITLE-ABS-KEY%28Influence+of+diet+on+the+gut+microbiome+and+implications+for+human+health.%29&relpos=0&citeCnt=1145&searchTerm=) | this review systematically evaluates current data regarding the effects of several common dietary components on intestinal microbiota. We show that consumption of particular types of food produces predictable shifts in existing host bacterial genera. Furthermore, the identity of these bacteria affects host immune and metabolic parameters, with broad implications for human health. Familiarity with these associations will be of tremendous use to the practitioner as well as the patient. |
| Diet rapidly and reproducibly alters the human gut microbiome. | David LA, Maurice CF, Carmody RN, Gootenberg DB, Button JE, Wolfe  BE, et al. | 2014 | [Nature. 2014;505:559–63.](https://www.nature.com/articles/nature12820) | An acute change in diet—for instance to one that is strictly animal-based or plant-based—alters microbial composition within just 24 h of initiation, with reversion to baseline within 48 h of diet discontinuation. Here we show that the short-term consumption of diets composed entirely of animal or plant products alters microbial community structure and overwhelms inter-individual differences in microbial gene expression. The animal-based diet increased the abundance of bile-tolerant microorganisms (Alistipes, Bilophila and Bacteroides) and decreased the levels of Firmicutes that metabolize dietary plant polysaccharides (Roseburia, Eubacterium rectale and Ruminococcus bromii). |
| Gut microbiota plasticity is correlated with sustained weight loss on a low-carb or low-fat dietary intervention | Grembi, J.A., Nguyen, L.H., Haggerty, T.D., (...), Holmes, S.P., Parsonnet, J. | 2020 | [Scientific Reports](https://link.springer.com/content/pdf/10.1038/s41598-020-58000-y.pdf)  [10(1),1405](https://link.springer.com/content/pdf/10.1038/s41598-020-58000-y.pdf) | We found no specifc bacterial signatures associated with weight loss that were consistent across both cohorts. However, the gut microbiota plasticity (i.e. variability), was correlated with long-term (12-month) weight loss in a diet-dependent manner; on the low-fat diet subjects with higher pre-diet daily plasticity had higher sustained weight loss, whereas on the low-carbohydrate diet those with higher plasticity over 10 weeks of dieting had higher 12-month weight loss. Our fndings suggest the potential importance of gut microbiota plasticity for sustained weight-loss. We highlight the advantages of evaluating kinetic trends and assessing reproducibility in studies of the gut microbiota |
| High-level adherence to a Mediterranean diet beneficially impacts the gut microbiota and associated metabolome. | De Filippis F, Pellegrini N, Vannini L, et al. | 2016 | *Gut*.2016;65(11):1812-1821. doi:10.1136/gutjnl-2015-309957 | We detected significant associations between consumption of vegetable-based diets and increased levels of faecal short-chain fatty acids, Prevotella and some fibre-degrading Firmicutes, whose role in human gut warrants further research. Conversely, we detected higher urinary trimethylamine oxide levels in individuals with lower adherence to the MD. Conclusions: High-level consumption of plant foodstuffs consistent with an MD is associated with beneficial microbiome-related metabolomic profiles in subjects ostensibly consuming a Western diet. |
| Health benefit of vegetable/fruit juice-based diet: Role of microbiome. | Henning SM, Yang J, Shao P, et al. | 2017 | *Sci Rep. 2017;7(1):2167. Published 2017 May 19. doi:10.1038/s41598-017-02200-6* | It was our hypothesis that changes in the intestinal microbiota induced by a juice-based diet play an important role in their health benefits. Twenty healthy adults consumed only vegetable/fruit juices for 3 days followed by 14 days of customary diet. On day 4 we observed a significant decrease in weight and body mass index (p = 2.0E-05), which was maintained until day 17 (p = 3.0E-04). On day 4 the proportion of the phylum Firmicutes and Proteobacteria in stool was significantly decreased and Bacteroidetes and Cyanobacteria was increased compared to baseline and was partially reversed on day 17. On day 4 plasma and urine nitric oxide was increased by 244 ± 89% and 450 ± 360%, respectively, and urinary lipid peroxidation marker malondialdehyde was decreased by 32 ± 21% compared to baseline. General well-being score was increased at the end of the study. In summary a 3-day juice-based diet altered the intestinal microbiota associated with weight loss, increase in the vasodilator NO, and decrease in lipid oxidation. |
| Gut Microbiome Composition in Non-human Primates Consuming a Western or Mediterranean Diet. | Nagpal R, Shively CA, Appt SA, et al. | 2018 | *Front Nutr. 2018;5:28. Published 2018 Apr 25. doi:10.3389/fnut.2018.00028* | Non-human primates, our close phylogenetic relatives and ancestors, provide an excellent model for studying diet-microbiome interaction; however, compared to clinical and rodent studies, research targeting primate gut microbiome has been limited. Herein, we analyze the gut microbiome composition in female cynomolgus macaques (Macaca fascicularis; n = 20) after the long-term (2.5 years) consumption of diets designed to mimic recent human Western- (WD; n = 10) or Mediterranean-type (MD; n = 10) diets. Microbiome diversity in MD consumers was significantly higher by the Shannon diversity index compared to the WD consumers, with similar but non-significant trends noted for the diversity metrics of species richness (Chao 1), observed operational taxonomic units (OTUs) and phylogenetic diversity (PD) whole Tree. Compared to the MD, the WD group demonstrated a higher Firmicutes-Bacteroides ratio and a significantly higher abundance of families Clostridiacea and Lactobacillaceae. Further analyses reveal significantly higher abundance of genera Lactobacillus, Clostridium, Faecalibacterium, and Oscillospira and lower abundance of Ruminococcus and Coprococcus in MD consumers relative to WD consumers. OTUs belonging to several species also show significant differences between the two groups, with Lactobacillus species demonstrating a prominently higher abundance in the MD consumers. |
| Consumption of Two Healthy Dietary Patterns Restored Microbiota Dysbiosis in Obese Patients with Metabolic Dysfunction. | Haro C, García-Carpintero S, Rangel-Zúñiga OA, et al. | 2017 | *Mol Nutr Food Res. 2017;61(12):10.1002/mnfr.201700300. doi:10.1002/mnfr.201700300* | Our results suggest that the chronic intake of two healthy dietary patterns partially restores the gut microbiome dysbiosis in obese patients with coronary heart disease, depending on the degree of metabolic dysfunction. |
| Dietary Fiber, Gut Microbiota, and Metabolic Regulation-Current Status in Human Randomized Trials. | Myhrstad MCW, Tunsjø H, Charnock C, Telle-Hansen VH. | 2020 | *Nutrients. 2020;12(3):859. Published 2020 Mar 23. doi:10.3390/nu12030859* | The aim of the present review is to summarize recent research on human randomized, controlled intervention studies investigating the effect of dietary fiber on gut microbiota and metabolic regulation. Metabolic regulation is discussed with respect to markers relating to glycemic regulation and lipid metabolism. Taken together, the papers on which the current review is based, suggest that dietary fiber has the potential to change the gut microbiota and alter metabolic regulation. However, due to the heterogeneity of the studies, a firm conclusion describing the causal relationship between gut microbiota and metabolic regulation remains elusive. |
| Gut Microbiota-Derived Short Chain Fatty Acids Induce Circadian Clock Entrainment in Mouse Peripheral Tissue. | Tahara Y, Yamazaki M, Sukigara H, et al. | 2018 | *Sci Rep. 2018;8(1):1395. Published 2018 Jan 23. doi:10.1038/s41598-018-19836-7* | Microbiota-derived short-chain fatty acids (SCFAs) and organic acids produced by the fermentation of non-digestible fibre can communicate from the microbiome to host tissues and modulate homeostasis in mammals. The microbiome has circadian rhythmicity and helps the host circadian clock function. We investigated the effect of SCFA or fibre-containing diets on circadian clock phase adjustment in mouse peripheral tissues (liver, kidney, and submandibular gland). Initially, caecal SCFA concentrations, particularly acetate and butyrate, induced significant day-night differences at high concentrations during the active period, which were correlated with lower caecal pH. By monitoring luciferase activity correlated with the clock gene Period2 in vivo, we found that oral administration of mixed SCFA (acetate, butyrate, and propionate) and an organic acid (lactate), or single administration of each SCFA or lactate for three days, caused phase changes in the peripheral clocks with stimulation timing dependency. However, this effect was not detected in cultured fibroblasts or cultured liver slices with SCFA applied to the culture medium, suggesting SCFA-induced indirect modulation of circadian clocks in vivo. Finally, cellobiose-containing diets facilitated SCFA production and refeeding-induced peripheral clock entrainment. SCFA oral gavage and prebiotic supplementation can facilitate peripheral clock adjustment, suggesting prebiotics as novel therapeutic candidates for misalignment. |
| Human and rat gut microbiome composition is maintained following sleep restriction. | Zhang SL, Bai L, Goel N, et al. | 2017 | *Proc Natl Acad Sci U S A. 2017;114(8):E1564-E1571. doi:10.1073/pnas.1620673114* | Insufficient sleep increasingly characterizes modern society, contributing to a host of serious medical problems. Loss of sleep is associated with metabolic diseases such as obesity and diabetes, cardiovascular disorders, and neurological and cognitive impairments. Shifts in gut microbiome composition have also been associated with the same pathologies; therefore, we hypothesized that sleep restriction may perturb the gut microbiome to contribute to a disease state. In this study, we examined the fecal microbiome by using a cross-species approach in both rat and human studies of sleep restriction. We used DNA from hypervariable regions (V1-V2) of 16S bacteria rRNA to define operational taxonomic units (OTUs) of the microbiome. Although the OTU richness of the microbiome is decreased by sleep restriction in rats, major microbial populations are not altered. Only a single OTU, TM7-3a, was found to increase with sleep restriction of rats. In the human microbiome, we find no overt changes in the richness or composition induced by sleep restriction. Together, these results suggest that the microbiome is largely resistant to changes during sleep restriction. |
| Gut microbiome diversity is associated with sleep physiology in humans. | Smith RP, Easson C, Lyle SM, et al. | 2019 | *PLoS One. 2019;14(10):e0222394. Published 2019 Oct 7. doi:10.1371/journal.pone.0222394* | The relationship between sleep physiology and the gut microbiome remains unclear. To address this uncertainty, we used actigraphy to quantify sleep measures coupled with gut microbiome sampling to determine how the gut microbiome correlates with various measures of sleep physiology. We measured immune system biomarkers and carried out a neurobehavioral assessment as these variables might modify the relationship between sleep and gut microbiome composition. We found that total microbiome diversity was positively correlated with increased sleep efficiency and total sleep time, and was negatively correlated with wake after sleep onset. We found positive correlations between total microbiome diversity and interleukin-6, a cytokine previously noted for its effects on sleep. Analysis of microbiome composition revealed that within phyla richness of Bacteroidetes and Firmicutes were positively correlated with sleep efficiency, interleukin-6 concentrations and abstract thinking. Finally, we found that several taxa (Lachnospiraceae, Corynebacterium, and Blautia) were negatively correlated with sleep measures. Our findings initiate linkages between gut microbiome composition, sleep physiology, the immune system and cognition. They may lead to mechanisms to improve sleep through the manipulation of the gut microbiome. |
| The shift work and health research agenda: Considering changes in gut microbiota as a pathway linking shift work, sleep loss and circadian misalignment, and metabolic disease. | Reynolds AC, Paterson JL, Ferguson SA, Stanley D, Wright KP Jr, Dawson D. | 2017 | *leep Med Rev. 2017;34:3-9. doi:10.1016/j.smrv.2016.06.009* | Recent findings have shown altered intestinal microbial communities and dysbiosis of the gut microbiota in circadian disrupted mice and jet lagged humans. We hypothesize that sleep and circadian disruption in humans alters the gut microbiota, contributing to an inflammatory state and metabolic disease associated with shift work. A research agenda for exploring the relationship between insufficient sleep, circadian misalignment and the gut microbiota is provided. |
| Gut microbiota and glucometabolic alterations in response to recurrent partial sleep deprivation in normal-weight young individuals. | Benedict C, Vogel H, Jonas W, et al. | 2016 | *Mol Metab. 2016;5(12):1175-1186. Published 2016 Oct 24. doi:10.1016/j.molmet.2016.10.003* | Microbiota composition analysis (V4 16S rRNA gene sequencing) revealed that after two days of PSD vs. after two days of NS, individuals exhibited an increased Firmicutes:Bacteroidetes ratio, higher abundances of the families Coriobacteriaceae and Erysipelotrichaceae, and lower abundance of Tenericutes (all P < 0.05) - previously all associated with metabolic perturbations in animal or human models. However, no PSD vs. NS effect on beta diversity or on fecal short-chain fatty acid concentrations was found. Fasting and postprandial insulin sensitivity decreased after PSD vs. NS (all P < 0.05). Discussion: Our findings demonstrate that short-term sleep loss induces subtle effects on human microbiota. To what extent the observed changes to the microbial community contribute to metabolic consequences of sleep loss warrants further investigations in larger and more prolonged sleep studies, to also assess how sleep loss impacts the microbiota in individuals who already are metabolically compromised. |
| Shorter sleep time relates to lower human defensin 5 secretion and compositional disturbance of the intestinal microbiota accompanied by decreased short-chain fatty acid production. | Shimizu Y, Yamamura R, Yokoi Y, et al. | 2023 | *Gut Microbes. 2023;15(1):2190306. doi:10.1080/19490976.2023.2190306* | Short sleep is known to increase disease risks via imbalance of intestinal microbiota, dysbiosis. However, mechanisms by which short sleep induces dysbiosis remain unknown. Small intestinal Paneth cell regulates the intestinal microbiota by secreting antimicrobial peptides including α-defensin, human defensin 5 (HD5). Disruption of circadian rhythm mediating sleep-wake cycle induces Paneth cell failure. We aim to clarify effects of short sleep on HD5 secretion and the intestinal microbiota. Fecal samples and self-reported sleep time were obtained from 35 healthy middle-aged Japanese (41 to 60-year-old). Shorter sleep time was associated with lower fecal HD5 concentration (r = 0.354, p = 0.037), lower centered log ratio (CLR)-transformed abundance of short-chain fatty acid (SCFA) producers in the intestinal microbiota such as [Ruminococcus] gnavus group (r = 0.504, p = 0.002) and Butyricicoccus (r = 0.484, p = 0.003), and lower fecal SCFA concentration. Furthermore, fecal HD5 positively correlated with the abundance of these genera and SCFA concentration. These findings suggest that short sleep relates to disturbance of the intestinal microbiota via decreased HD5 secretion. |
| Clarithromycin in γ-aminobutyric acid-Related hypersomnolence: A randomized, crossover trial. | Trotti LM, Saini P, Bliwise DL, Freeman AA, Jenkins A, Rye DB. | 2015 | *Ann Neurol. 2015;78(3):454-465. doi:10.1002/ana.24459* | Subjective sleepiness, but not psychomotor vigilance, improved during a 2-week course of clarithromycin. Although additional studies are needed, this suggests that clarithromycin may be a reasonable treatment option in patients with treatment-refractory hypersomnolence. This trial was registered at ClinicalTrials.gov (NCT01146600) and supported by the American Sleep Medicine Foundation. |
| Beneficial effects of Lactobacillus casei strain Shirota on academic stress-induced sleep disturbance in healthy adults: a double-blind, randomised, placebo-controlled trial | Takada M, Nishida K, Gondo Y, Kikuchi-Hayakawa H, Ishikawa H, Suda K, et al. | 2017 | [Beneficial Microbes, 8(2), pp. 153–162](https://www.scopus.com/record/display.uri?eid=2-s2.0-85018329668&origin=resultslist&sort=plf-f&src=s&sid=9494703f622f541fd2c0b8a9e7b6e681&sot=b&sdt=b&s=TITLE-ABS-KEY%28Beneficial++effects+of+Lactobacillus+casei+strain+Shirota+on+academic+stress-induced+sleep+disturbance+in++healthy+adults%3A+a+double-blind%2C+randomised%2C+placebo-controlled+trial.%29&sl=105&sessionSearchId=9494703f622f541fd2c0b8a9e7b6e681) | There was a significant positive effect of Lactobacillus casei strain Shirota (LcS) treatment on Oguri-Shirakawa-Azumi (OSA) sleep inventory scores of subjective sleep quality factors for sleepiness on rising and sleep length. Sleep latency measured by EEG lengthened as the exam approached in the placebo group but was significantly suppressed in the LcS group. The percentage of stage 3 non-REM (N3) sleep decreased in the placebo group as the exam approached, whereas it was maintained in the LcS group throughout the trial. Delta power during the first sleep cycle, measured as an index of sleep intensity, increased as the exam approached in the LcS group and was significantly higher than in the placebo group. These findings suggest that daily consumption of LcS may help to maintain sleep quality during a period of increasing stress. The observed retention of N3 sleep and increased delta power in the LcS group may have contributed to higher perceived sleep satisfaction |

| Document title | Authors | Year | Source | Results, conclusions |
| --- | --- | --- | --- | --- |
| Tryptophan-enriched cereal intake improves nocturnal sleep, melatonin, serotonin, and total antioxidant capacity levels and mood in elderly humans | Bravo, R., Matito, S., Cubero, J., Paredes, S.D., Franco, L., Rivero, M., Rodríguez, A.B., (...), Barriga, C. | 2013 | [Age, 35 (4), pp. 1277-1285](https://www.scopus.com/record/display.uri?eid=2-s2.0-84891717197&origin=reflist&sort=r-f&src=s&st1=sleep+and+diet%3a+mounting&nlo=&nlr=&nls=&sid=3b72eb5df3cca5f1d54b130d6dd7195e&sot=b&sdt=b&sl=39&s=TITLE-ABS-KEY%28sleep+and+diet%3a+mounting%29) | The consumption of cereals containing the higher dose in tryptophan increased sleep efficiency, actual sleep time, immobile time, and decreased total nocturnal activity, sleep fragmentation index, and sleep latency. Urinary 6-sulfatoxymelatonin, 5- hydroxyindoleacetic acid levels, and urinary total antioxidant capacity also increased respectively after tryptophan-enriched cereal ingestion as well as improving anxiety and depression symptoms. Cereals enriched with tryptophan may be useful as a chrononutrition tool for alterations in the sleep/wake cycle due to age |
| Post-prandial changes in measures of fatigue: Effect of a mixed or a pure carbohydrate or pure fat meal | Cunliffe, A., Obeid, O.A., Powell-Tuck, J. | 1997 | [European Journal of Clinical Nutrition](https://www.nature.com/articles/1600496)  [51(12), pp. 831-838](https://www.nature.com/articles/1600496) | Conclusions: Central and subjective fatigue may be influenced by raised plasma free tryptophan to competitor amino acid ratios induced by carbohydrate intake but other aspects of central arousal are affected by fat intake. |
| Sleep and Diet: Mounting Evidence of a Cyclical Relationship | Zuraikat, F.M., Wood, R.A., Barragán, R., St-Onge, M.-P. | 2021 | [Annual Review of Nutrition](https://www.scopus.com/redirect/linking.uri?targetURL=https%3a%2f%2fdoi.org%2f10.1146%2fannurev-nutr-120420-021719&locationID=2&categoryID=4&eid=2-s2.0-85117419327&issn=01999885&linkType=ViewAtPublisher&year=2021&origin=resultslist&dig=4a1292a12ac0a588c66b372a4e4e7d63)  [41, pp. 309-332](https://www.scopus.com/redirect/linking.uri?targetURL=https%3a%2f%2fdoi.org%2f10.1146%2fannurev-nutr-120420-021719&locationID=2&categoryID=4&eid=2-s2.0-85117419327&issn=01999885&linkType=ViewAtPublisher&year=2021&origin=resultslist&dig=4a1292a12ac0a588c66b372a4e4e7d63) | diet may influence sleep via melatonin and its biosynthesis from tryptophan. Experimental data exist indicating that provision of specific foods rich in tryptophan or melatonin can improve sleep quality. Whole diets rich in fruits, vegetables, legumes, and other sources of dietary tryptophan and melatonin have been shown to predict favorable sleep outcomes. Although clinical trials are needed to confirm a causal impact of dietary patterns on sleep and elucidate underlying mechanisms, available data illustrate a cyclical relation between these lifestyle factors. We recommend adopting a healthful diet to improve sleep, which may further promote sustained favorable dietary practices. |
| Effects of Diet on Sleep: A Narrative Review | Binks, H., Vincent, G.E., Gupta, C., Irwin, C., Khalesi, S. | 2020 | [Nutrients](https://www.mdpi.com/2072-6643/12/4/936)  [12(4),936](https://www.mdpi.com/2072-6643/12/4/936) | Four themes emerged: tryptophan consumption and tryptophan depletion, dietary supplements, food items, and macronutrients. High carbohydrate diets, and foods containing tryptophan, melatonin, and phytonutrients (e.g., cherries), were linked to improved sleep outcomes. The authors posit that these effects may be due in part to dietary influences on serotonin and melatonin activity |
| Diet promotes sleep duration and quality | Peuhkuri, K., Sihvola, N., Korpela, R. | 2012 | [Nutrition Research](https://linkinghub.elsevier.com/retrieve/pii/S0271531712000632)  [32(5), pp. 309-319](https://linkinghub.elsevier.com/retrieve/pii/S0271531712000632) | Overall, foods impacting the availability of tryptophan, as well as the synthesis of serotonin and melatonin, may be the most helpful in promoting sleep. Although there are clear physiological connections behind these effects, the clinical relevance needs to be studied further. |
| A dietary supplement to improve the quality of sleep: A randomized placebo controlled trial | Cornu, C., Remontet, L., Noel-Baron, F., (...), Saadatian-Elahi, M., Kassaï, B. | 2010 | [BMC Complementary and Alternative Medicine](https://bmccomplementmedtherapies.biomedcentral.com/articles/10.1186/1472-6882-10-29)  [10,29](https://bmccomplementmedtherapies.biomedcentral.com/articles/10.1186/1472-6882-10-29) | The average of Leeds score was similar in both groups (p = 0.95). A marked improvement in the quality of sleep was observed in both placebo (62%) and active (65%) group (p = 0.52). The evolution of urinary melatonin, aMT6S, and of the Mel/aMT6S ratio showed no differences between the two groups. Sleep efficiency, as measured by actigraphy, improved similarly in both groups during the treatment period, from 72% to 76% and 75% in the active and placebo group respectively (p = 0.91).Conclusions: The dietary supplement had neither effect on the perceived quality of sleep, nor on the melatonin metabolism and sleep-wake cycle. |
| Melatonin, serotonin, and tryptamine in some Egyptian food and medicinal plants | Badria, F.A. | 2002 | [Journal of Medicinal Food, 5 (3), pp. 153-157](https://www.scopus.com/record/display.uri?eid=2-s2.0-0036754454&origin=reflist&sort=r-f&src=s&st1=sleep+and+diet%3a+mounting&nlo=&nlr=&nls=&sid=3b72eb5df3cca5f1d54b130d6dd7195e&sot=b&sdt=b&sl=39&s=TITLE-ABS-KEY%28sleep+and+diet%3a+mounting%29) | Three substances that are very important in animal physiology (e.g., in brain metabolism) are noradrenaline, histamine, and serotonin; all three occur in plants. HThe results of this screening showed that the pulp of underripe and ripe yellow banana contains 5-hydroxytryptamine at concentrations of 31.4 and 18.5 ng/g, respectively. Corn, rice, barley grains, and ginger showed the highest concentrations of melatonin, at 187.8, 149.8, 87.3, 142.3 ng/100 g, respectively. On the other hand, potato samples were free from all indolamines. Pomegranate and strawberry showed a low level of indolamines (8-12 μg/g serotonin, 4-9 μg/g tryptamine, and 13-29 ng/100 g melatonin). |
| Functional roles of melatonin in plants, and perspectives in nutritional and agricultural science. | Tab DX, Hardeland R. Manchester LC, Korkmaz A, Ma S. Rosales-Corral S, Reiter RJ. | 2012 | J Exp Bot. 2012 Jan;63(2):577-97 | Importantly, remarkably high melatonin concentrations have been measured in popular beverages (coffee, tea, wine, and beer) and crops (corn, rice, wheat, barley, and oats). Billions of people worldwide consume these products daily. The beneficial effects of melatonin on human health derived from the consumption of these products must be considered. Evidence also indicates that melatonin has an ability to increase the production of crops. The mechanisms may involve the roles of melatonin in preservation of chlorophyll, promotion of photosynthesis, and stimulation of root development. Transgenic plants with enhanced melatonin content could probably lead to breakthroughs to increase crop production in agriculture and to improve the general health of humans. |
| *Effects of exogenous melatonin on sleep: a meta-analysis.* | Brzezinski A, Vangel MG, Wurtman RJ, et al. | 2005 | *Sleep Med Rev*. 2005;9(1):41-50. doi:10.1016/j.smrv.2004.06.004 | Sleep onset latency, total sleep duration, and sleep efficiency were selected as the outcome measures. The study effect size was taken to be the difference between the response on placebo and the mean response on melatonin for each outcome measured. Melatonin treatment significantly reduced sleep onset latency by 4.0 min (95% CI 2.5, 5.4); increased sleep efficiency by 2.2% (95% CI 0.2, 4.2), and increased total sleep duration by 12.8 min (95% CI 2.9, 22.8). Since 15 of the 17 studies enrolled healthy subjects or people with no relevant medical condition other than insomnia, the analysis was also done including only these 15 studies. The sleep onset results were changed to 3.9 min (95% CI (2.5, 5.4)); sleep efficiency increased to 3.1% (95% CI (0.7, 5.5)); sleep duration increased to 13.7 min (95% CI (3.1, 24.3)). |

| Document title | Authors | Year | Source | Results, conclusions |
| --- | --- | --- | --- | --- |
| Dietary inflammatory index and sleep quality in Southern Italian adults | Godos, J., Ferri, R., Caraci, F., (...), Galvano, F., Grosso, G. | 2019 | Nutrients  11(6),1324 | Results: Individuals in the highest quartile of the (dietary inflammatory index) DII were less likely to have adequate sleep quality. Among individual domains of sleep quality, an association with the highest exposure category was found only for sleep latency . Conclusions: The inflammatory potential of the diet appears to be associated with sleep quality in adults. Interventions to improve diet quality might consider including a dietary component that aims to lower chronic systemic inflammation to prevent cognitive decline and improve sleep quality. |
| Oxidative stress in patients with primary insomnia | Gulec, M., Ozkol, H., Selvi, Y., (...), Besiroglu, L., Ozdemir, P.G. | 2012 | Progress in Neuro-Psychopharmacology and Biological Psychiatry  37(2), pp. 247-251 | Results: Our results show that the patients with primary insomnia had significantly lower GSH-Px activity and higher MDA levels compared with the controls. Conclusion: These results may indicate the important role of sleep in attenuating oxidative stress. |
| Sleep disturbance, sleep duration, and inflammation: A systematic review and meta-analysis of cohort studies and experimental sleep deprivation | Irwin, M.R., Olmstead, R., Carroll, J.E. | 2016 | [Biological Psychiatry](https://www-sciencedirect-com.rossuniversity.idm.oclc.org/science/article/abs/pii/S0006322315004370?via%3Dihub)  [80(1), pp. 40-52](https://www-sciencedirect-com.rossuniversity.idm.oclc.org/science/article/abs/pii/S0006322315004370?via%3Dihub) | A total of 72 studies (n > 50,000) were analyzed with assessment of C-reactive protein (CRP), interleukin-6 (IL-6), and tumor necrosis factor α (TNFα). Sleep disturbance was associated with higher levels of CRP and IL-6 . Shorter sleep duration, but not the extreme of short sleep, was associated with higher levels of CRP but not IL-6. The extreme of long sleep duration was associated with higher levels of CRP and IL-6. Neither sleep disturbances nor sleep duration was associated with TNFα. Neither experimental sleep deprivation nor sleep restriction was associated with CRP, IL-6, or TNFα. |
| Overall diet quality and proinflammatory diet in relation to risk of obstructive sleep apnea in 3 prospective US cohorts. | Liu Y, Tabung FK, Stampfer MJ, Redline S, Huang T. | 2022 | *Am J Clin Nutr*. 2022;116(6):1738-1747. doi:10.1093/ajcn/nqac257 | We documented 8856 incident OSA cases during follow-up. In pooled analyses adjusted for potential confounders, higher diet quality (higher AHEI scores) was associated with lower OSA risk (HR comparing the highest with the lowest quintile of AHEI: 0.76; 95% CI: 0.71, 0.82; P-trend < 0.001), and higher dietary inflammatory potential (higher EDIP scores) was associated with significantly increased risk (HR comparing the highest with the lowest quintile of EDIP: 1.94; 95% CI: 1.81, 2.08; P-trend < 0.001). The association with AHEI score was no longer statistically significant (comparable HR: 0.98; 95% CI: 0.91, 1.05; P-trend = 0.54), whereas the association with EDIP score remained statistically significant (comparable HR: 1.31; 95% CI: 1.22, 1.41; P-trend < 0.001). Conclusions: A healthier diet, particularly one with anti-inflammatory potential, was associated with lower OSA risk. |

| Document title | Authors | Year | Source | Results, conclusions |
| --- | --- | --- | --- | --- |
| Relationship between daily  isoflavone intake and sleep in Japanese adults: a cross-sectional study. | Cui Y, Niu K, Huang C, Momma H, Guan L, Kobayashi Y, et al. | 2015 | [Nutrition Journal](https://nutritionj.biomedcentral.com/articles/10.1186/s12937-015-0117-x)  [14(1),127](https://nutritionj.biomedcentral.com/articles/10.1186/s12937-015-0117-x) | In the present study, higher daily isoflavone intake from food was significantly associated with better sleep duration and higher sleep quality in a Japanese population, even after adjusting for potentially confounding factors. This suggests that the daily intake of isoflavones in food may have a potentially beneficial effect on sleep duration and quality, Prospective or interventional studies are required to clarify the causality. |
| Isoflavones decrease insomnia in postmenopause | Hachul H, Brandao LC, D’Almeida V, Bittencourt LR, Baracat EC, Tufik S | 2011 | [Menopause. 2011;18:178–84.](https://www.scopus.com/record/display.uri?eid=2-s2.0-79951578517&origin=resultslist&sort=r-f&src=s&st1=Isoflavones+decrease+insomnia+in+postmenopause&nlo=&nlr=&nls=&sid=9d16d3300c57c80ddb5bc671e1336c8d&sot=b&sdt=b&sl=61&s=TITLE-ABS-KEY%28Isoflavones+decrease+insomnia+in+postmenopause%29&relpos=0&citeCnt=43&searchTerm=) | Results: Thirty-eight women were enrolled in the study. Polysomnography revealed a significant increase in sleep efficiency in the isoflavone group (from 77.9% to 83.9%) when compared with the placebo group (from 77.6% to 81.2%). Isoflavones induced a decrease in the intensity and number of hot flashes and the frequency of insomnia: among the women in the placebo group, 94.7% had moderate or intense insomnia at the beginning of the study, compared with 63.2% at the end, whereas in the isoflavone group, these percentages were 89.5% and 36.9%, respectively. Conclusions: In postmenopausal women with insomnia, isoflavone treatment was effective in reducing insomnia symptoms, which was confirmed by increased sleep efficiency as observed by polysomnographic analysis. |
| The pros and cons of plant estrogens for menopause | Bedell, S., Nachtigall, M., Naftolin, F. | 2014 | [Journal of Steroid Biochemistry and Molecular Biology](https://www.sciencedirect.com/science/article/pii/S0960076012002567?via%3Dihub)  [139, pp. 225-236](https://www.sciencedirect.com/science/article/pii/S0960076012002567?via%3Dihub) | This review scrutinizes the most current research regarding the efficacy of three types of phytoestrogens, isoflavones, lignans and coumestans, and their specific effect on the reduction of climacteric symptoms, specifically vasomotor symptoms, vaginal atrophy, insomnia and osteoporosis. Certain phytoestrogens have also been shown to decrease vaginal atrophy, improve sleep and cognition, and positively affect bone health. |
| Soy Isoflavone Intake and Sleep Parameters over 5 Years among Chinese Adults: Longitudinal Analysis from the Jiangsu Nutrition Study | Cao Y, Taylor AW, Zhen S, Adams R, Appleton S, Shi Z. Soy | 2017 | [Journal of the Academy of Nutrition and Dietetics. 2017; 117(4):536–44e2.](https://www.sciencedirect.com/science/article/pii/S2212267216312989?via%3Dihub) | The prevalence of long sleep duration was 18.9% in 2002 and 12.6% in 2007, and the prevalence of daytime falling asleep was 5.3%. Compared with the lowest quartile of isoflavone intake, the highest quartile was associated with a lower risk of long sleep duration (odds ratio=0.66; 95% CI 0.48 to 0.90; P for trend=0.018) over 5 years. Compared with persistent low intake of isoflavone (less than median intake of isoflavone at two time points), persistent high intake was associated with a reduced risk of daytime falling asleep in women (odds ratio=0.20; 95% CI 0.06 to 0.68), but not men. No consistent association between soy isoflavone intake and short sleep duration was found. Conclusions: Soy isoflavone intake was associated with a low risk of long sleep duration in both sexes and a low risk of daytime falling asleep in women but not men. |

| Document title | Authors | Year | Source | Results, conclusions |
| --- | --- | --- | --- | --- |
| Effect of an Interdisciplinary Weight Loss and Lifestyle Intervention on Obstructive Sleep Apnea Severity  The INTERAPNEA Randomized Clinical Trial | Carneiro-Barrera, A., Amaro-Gahete, F.J.,Guillén-Riquelme, A., ...Buela-Casal, G., Ruiz, J.R. | 2022 | [JAMA Network Open, 5(4), pp. E228212](https://jamanetwork.com/journals/jamanetworkopen/fullarticle/2791455) | 89 Spanish men with moderate to severe OSA who had overweight or obesity and were receiving CPAP therapy, an 8-week interdisciplinary weight loss and lifestyle intervention significantly improved OSA severity and other outcomes compared with usual care alone. At 8 weeks, 45% of participants in the intervention group no longer required CPAP therapy; at 6 months, 62% of participants in the intervention group no longer required CPAP therapy. |
| Progression and regression of sleep-disordered breathing with changes in weight: The Sleep Heart Health Study | Newman, A.B., Foster, G., Givelber, R., (...), Redline, S., Young, T. | 2005 | [Archives of Internal Medicine 165(20), pp. 2408-2413](https://jamanetwork-com.rossuniversity.idm.oclc.org/journals/jamainternalmedicine/fullarticle/486784) | Both men and women had a greater increase in Respiratory Disturbance Index (RDI) with weight gain than a decrease in RDI with weight loss. Conclusion: Modest changes in weight were related to an increase or decrease in sleep-disordered breathing (SDB). |
| Obesity without sleep apnoea is associated with daytime sleepiness. | A N Vgontzas 1 , E O Bixler, T L Tan, D Kantner, L F Martin, A Kales | 1998 | [Archives of Internal Medicine 158(12), pp. 1333-1337](https://jamanetwork-com.rossuniversity.idm.oclc.org/journals/jamainternalmedicine/fullarticle/207201) | Daytime sleepiness and fatigue are frequent complaints of obese patient even among those who do not demonstrate sleep apnoea. It was shown that the percentage of sleep time in the daytime was significantly higher in obese patients, whereas in the night-time the percentage of sleep time was lower among the obese subjects. An analysis of the relationship between nighttime and daytime sleep suggested that daytime sleepiness in obese patients is a result of a circadian abnormality rather than just being secondary to night-time sleep disturbance. |
| Two year reduction in sleep apnea symptoms and associated diabetes incidence after weight loss in severe obesity | Grunstein, R.R., Stenlöf, K., Hedner, J.A., (...), Karason, K., Sjöström, L. | 2000 | [Sleep 30(6), pp. 703-710](https://academic-oup-com.rossuniversity.idm.oclc.org/sleep/article/30/6/703/2696753) | Bariatric surgery results in a marked improvement in sleep apnea symptoms at 2 years. |
| Weight loss as a treatment for obstructive sleep apnoea | Barvaux, V.A., Aubert, G., Rodenstein, D.O. | 2000 | [Sleep Medicine Reviews, Vol. 4, No. 5, pp 435–452](https://www-sciencedirect-com.rossuniversity.idm.oclc.org/science/article/abs/pii/S108707920090114X?via%3Dihub) | Weight loss is essential in order to reduce the severity of OSA. Weight loss alone rarely cures OSA; classical techniques of treatment of OSA, such as nCPAP can be used as a combined therapy, such as nCPAP can be used as a combined therapy |
| Gastric bypass is an effective treatment for obstructive sleep apnea in patients with clinically significant obesity | Rasheid, S., Banasiak, M., Gallagher, S.F., (...), Anderson, W.M., Murr, M.M. | 2003 | [Obesity Surgery 13(1), pp. 58-61](https://link-springer-com.rossuniversity.idm.oclc.org/article/10.1381/096089203321136593) | Weight loss following gastric bypass results in profound improvement in OSA.The severity of apnea cannot be reliably predicted by preoperative BMI and ESS; therefore, patients with symptoms of OSA should undergo polysomnography. |
| A randomized controlled study to examine the effect of a lifestyle modification program in OSA | Ng, Susanna S. S.a; Chan, Ruth S. M.a, b; Woo, Jeanb; Chan, Tat-Onc; Cheung, Bernice H. K.b; Sea, Mandy M. M.b; To, Kin-Wanga; Chan, Ken K. P.a; Ngai, Jennya; Yip, Wing-Hoa; Ko, Fanny W. S.a; Hui, David S. C.a | 2015 | [Chest 148(5), pp. 1193-1203](https://www-sciencedirect-com.rossuniversity.idm.oclc.org/science/article/abs/pii/S0012369215502304?via%3Dihub) | Eating behavior was improved with increased intake of protein and fiber. Dietecian-led lifestyle modification program (LMP) was effective in reducing the severity of OSA and daytime sleepiness. The beneficial effect was sustained in 12 months. |
| Effect of Weight Loss and Continuous Positive Airway Pressure on Obstructive Sleep Apnea and Metabolic Profile Stratified by Craniofacial Phenotype A Randomized Clinical Trial | Ng, S.S.S., Tam, W.W.S., Lee, R.W.W., (...), Cistulli, P.A., Hui, D.S. | 2020 | [American Journal of Respiratory and Critical Care Medicine 205(6), pp. 711-720](https://www.atsjournals.org/doi/10.1164/rccm.202106-1401OC) | Weight reduction alleviated subclinical inflammation and improved insulin sensitivity more than CPAP among obese patients with moderate to severe OSA, and this effect was not influenced by craniofacial structure. |
| Sleep disturbance and obesity: Changes following surgically induced weight loss | Dixon, J.B., Schachter, L.M., O'Brien, P.E. | 2001 | [Archives of Internal Medicine 161(1), pp. 102-106](https://jamanetwork-com.rossuniversity.idm.oclc.org/journals/jamainternalmedicine/fullarticle/646920) | Obesity-related sleep disorders improve markedly with weight loss. |
| Excess weight and sleep-disordered breathing | Young, T., Peppard, P.E., Taheri, S. | 2000 | [Journal of Applied Physiology 99(4), pp. 1592-1599](https://journals-physiology-org.rossuniversity.idm.oclc.org/doi/full/10.1152/japplphysiol.00587.2005) | ∼17% of adults have mild or worse SDB (apnea-hypopnea index ≥ 5) and that 41% of those adults have SDB " attributable" to having a body mass index of ≥25 kg/m2. Similarly, we estimate that -5.7% of adults have moderate or worse SDB (apnea-hypopnea index ≥ 15) and that 58% of those adults have SDB attributable to excess weight. |
| Obesity-related sleepiness and fatigue: The role of the stress system and cytokines | Vgontzas, A.N., Bixler, E.O., Chrousos, G.P. | 2006 | [Annals of the New York Academy of Sciences 1083, pp. 329-344](https://nyaspubs-onlinelibrary-wiley-com.rossuniversity.idm.oclc.org/doi/abs/10.1196/annals.1367.023) | Obesity-related objective daytime sleepiness and fatigue  are associated primarily with metabolic and psychological factors and  less with sleep apnea and sleep disruption per se. We suggest  that objective sleepiness is primarily related to metabolic factors,  whereas fatigue appears to be related to psychological distress. |
| Daytime sleepiness in the obese: Not as simple as obstructive sleep apnea | Dixon, J.B., Dixon, M.E., Anderson, M.L., Schachter, L., O'Brien, P.E. | 2007 | [Obesity 15(10), pp. 2504-2511](https://onlinelibrary-wiley-com.rossuniversity.idm.oclc.org/doi/full/10.1038/oby.2007.297) | In severely obese subjects, increased daytime sleepiness does not seem to be driven by obstructive sleep apnea, the degree of obesity, or anthropometric, metabolic, or inflammatory markers of the metabolic syndrome. It is, however, associated with poor energy, symptoms of depression, and symptoms of nocturnal sleep disturbance. |
| Cross-sectional relationship of reported fatigue to obesity, diet, and physical activity: Results from the Third National Health and Nutrition Examination Survey | Resnick, H.E., Carter, E.A., Aloia, M., Phillips, B. | 2006 | [Journal of Clinical Sleep Medicine 2(2), pp. 163-169](https://jcsm.aasm.org/doi/10.5664/jcsm.26511) | In adults aged 20 to 59 years in the United States, self-reported fatigue is associated with higher body mass index, higher waist circumference, and a reduced likelihood of getting recommended levels of physical activity. |
| A randomized study on the effect of weight loss on obstructive sleep apnea among obese patients with type 2 diabetes: The sleep AHEAD study | Foster, G.D., Borradaile, K.E., Sanders, M.H., (...), Reboussin, D., Kuna, S.T. | 2009 | [Archives of Internal Medicine 169(17), pp. 1619-1626](https://jamanetwork-com.rossuniversity.idm.oclc.org/journals/jamainternalmedicine/fullarticle/224770) | Physicians and their patients can expect that weight loss will result in significant and clinically relevant improvements in OSA among obese patients with type 2 diabetes. Participants with a weight loss of 10 kg or more had the greatest reductions in apnea-hypopnea index (AHI). |
| Resolution of obstructive sleep apnea after laparoscopic gastric bypass | Varela, J.E., Hinojosa, M.W., Nguyen, N.T. | 2007 | [Obesity Surgery 17(10), pp. 1279-1282](https://link-springer-com.rossuniversity.idm.oclc.org/article/10.1007/s11695-007-9228-6) | Weight loss associated with laparoscopic Roux-en- Y gastric bypass significantly improves the symptoms of sleep apnea and is effective in discontinuation in the clinical use of CPAP therapy. Improvement of obstructive sleep apnea symptoms occur as early as 1 month postoperatively. |
| Effects of weight reduction therapy on obstructive sleep apnea syndrome and arterial stiffness in patients with obesity and metabolic syndrome | Iguchi, A., Yamakage, H., Tochiya, M., (...), Shimatsu, A., Satoh-Asahara, N. | 2013 | [Journal of Atherosclerosis and Thrombosis 20(11), pp. 807-820](https://www.jstage.jst.go.jp/article/jat/20/11/20_17632/_article) | This study demonstrated that the severity of OSA is significantly correlated with the severity of Metabolic Syndrome and arterial stiffness in obese patients. Short-term weight reduction therapy improves not only metabolic dysfunction, but also the severity of OSA and arterial stiffness. |
| Daytime Sleepiness in Obesity: Mechanisms Beyond Obstructive Sleep Apnea—A Review | Panossian, L. A., Veasey, S. C. | 2012 | [Sleep, Volume 35, Issue 5, 1 May 2012, Pages 605–615,](https://academic.oup.com/sleep/article/35/5/605/2595926?login=false) | Both obesity and diet can directly contribute to sleepiness. Research in this field, as summarized in this review, has begun to elucidate the complex interrelationships between circulating systemic hormones and neuronal signaling pathways in the CNS. While studies have successfully identified candidate molecules for hypersomnolence in obesity, additional studies are needed to more firmly establish key molecular mechanisms involved, to discern acute and chronic effects of feeding and obesity |
| Obesity as an independent predictor of subjective excessive daytime sleepiness | Slater, G., Pengo, M.F., Kosky, C., Steier, J. | 2013 | [Respiratory Medicine](https://www-sciencedirect-com.rossuniversity.idm.oclc.org/science/article/pii/S0954611112003964?via%3Dihub)  [107(2), pp. 305-309](https://www-sciencedirect-com.rossuniversity.idm.oclc.org/science/article/pii/S0954611112003964?via%3Dihub) | Regression analysis revealed that obesity (p = 0.007), PLM disorder (p = 0.010) and hypertension (p = 0.032) were independently associated with subjective sleepiness (adjusted R2 = 0.384, p = 0.02), obesity accounting for 15.7% of the variability in sleepiness.Conclusion: Independent of underlying sleep disorders, obesity contributes significantly to daytime sleepiness. |
| Sleep is increased by weight gain and decreased by weight loss in mice | Guan, Z., Vgontzas, A.N., Bixler, E.O., Fang, J. | 2008 | [Sleep](https://academic.oup.com/sleep/article/31/5/627/2454193?login=true)  [31(5), pp. 627-633](https://academic.oup.com/sleep/article/31/5/627/2454193?login=true) | These observations indicate that sleep alterations induced by weight gain are reversed by weight loss in obese animals. (The weight gain/loss group displayed a significant decrease in wakefulness and increases in NREMS and episodes of NREMS during 6 weeks of weight gain, which were reversed during subsequent 4 weeks of weight loss.) |
| Longitudinal study of moderate weight change and sleep-disordered breathing | Peppard, P.E., Young, T., Palta, M., Dempsey, J., Skatrud, J. | 2020 | [JAMA](https://jamanetwork-com.rossuniversity.idm.oclc.org/journals/jama/fullarticle/193382)  [284(23), pp. 3015-3021](https://jamanetwork-com.rossuniversity.idm.oclc.org/journals/jama/fullarticle/193382) | Relative to stable weight, a 10% weight gain predicted an approximate 32% increase in the apnea-hypopnea index (AHI). A 10% weight loss predicted a 26% decrease in the AHI. A 10% increase in weight predicted a 6-fold (95% CI, 2.2-17.0) increase in the odds of developing moderate-to-severe SDB. Conclusions: Our data indicate that clinical and public health programs that result in even modest weight control are likely to be effective in managing SDB and reducing new occurrence of SDB. |
| Low sleep quality and daytime sleepiness in obese patients without obstructive sleep apnoea syndrome | Resta, O., Foschino Barbaro, M.P., Bonfitto, P., (...), Pannacciulli, N., De Pergola, G. | 2003 | [Journal of Internal Medicine](https://onlinelibrary-wiley-com.rossuniversity.idm.oclc.org/doi/full/10.1046/j.1365-2796.2003.01133.x)  [253(5), pp. 536-543](https://onlinelibrary-wiley-com.rossuniversity.idm.oclc.org/doi/full/10.1046/j.1365-2796.2003.01133.x) | his study clearly shows that severe obesity, even in the absence of OSAS, is associated with sleep-related disorders and excessive daytimy sleepiness (EDS). |
| Sleep duration and excessive daytime sleepiness are associated with obesity independent of diet and physical activity | Maugeri, A., Medina-Inojosa, J.R., Kunzova, S., (...), Geda, Y.E., Vinciguerra, M. | 2018 | [Nutrients](https://pubmed.ncbi.nlm.nih.gov/30177634/)  [10(9),1219](https://pubmed.ncbi.nlm.nih.gov/30177634/) | EDS was associated with greater odds of central obesity (OR = 1.72; 95%CI = 1.06⁻2.79; p = 0.030) |
| Effect of a very low energy diet on moderate and severe obstructive sleep apnoea in obese men: a randomised controlled trial | Johansson K, Neovius M, Lagerros YT, et al. | 2009 | [BMJ. 2009;339:b4609.](https://www.ncbi.nlm.nih.gov/pmc/articles/PMC2788899/) | Conclusion: Treatment with a low energy diet improved obstructive sleep apnoea in obese men, with the greatest effect in patients with severe disease. Long term treatment studies are needed to validate weight loss as a primary treatment strategy for obstructive sleep apnoea. |
| The effect of exercise on obstructive sleep apnea: a randomized and controlled trial. | Sengul YS, Ozalevli S, Oztura I, Itil O, Baklan B. | 2011 | [Sleep Breath. 2011;15:49–56.](https://academic.oup.com/sleep/article/34/12/1631/2454593?login=true) | Conclusions: Exercise appears not to change anthropometric characteristics and respiratory functions while it improves AHI, health-related quality of life, quality of sleep, and exercise capacity in the patients with mild to moderate OSAS. |
| Lifestyle intervention with weight reduction: first-line treatment in mild obstructive sleep apnea. | Tuomilehto, H.P.I., Seppä, J.M., Partinen, M.M., (...), Tukiainen, H., Uusitupa, M. | 2009 | [American Journal of Respiratory and Critical Care Medicine](https://www.atsjournals.org/doi/10.1164/rccm.200805-669OC)  [179(4), pp. 320-327](https://www.atsjournals.org/doi/10.1164/rccm.200805-669OC) | Conclusions: a very low calorie diet (VLCD) combined with active lifestyle counseling resulting in marked weight reduction is a feasible and effective treatment for the majority of patients with mild OSA, and the achieved beneficial outcomes are maintained at 1-year follow-up. |
| Longer term effects of very low energy diet on obstructive sleep apnoea in cohort derived from randomised controlled trial: prospective observational follow-up study. | Johansson, K., Hemmingsson, E., Harlid, R., (...), Rössner, S., Neovius, M. | 2011 | [BMJ](https://www.bmj.com/content/342/bmj.d3017)  [342(7809),d3017](https://www.bmj.com/content/342/bmj.d3017) | Conclusion: Initial improvements in obstructive sleep apnoea after treatment with a very low energy diet can be maintained after one year in obese men with moderate to severe disease. Those who lose the most weight or have severe sleep apnoea at baseline benefit most. |
| Effectiveness of Lifestyle Interventions on Obstructive Sleep Apnea (OSA): Systematic Review and Meta-Analysis | Araghi, M.H., Chen, Y.-F., Jagielski, A., (...), Neil Thomas, G., Taheri, S. | 2013 | [Sleep. 2013 Oct 1; 36(10): 1553–1562.](https://www.ncbi.nlm.nih.gov/pmc/articles/PMC3773205/) | Results: Seven randomized controlled trials (519 participants) showed that weight reduction programs were associated with a decrease in AHI . Nine uncontrolled before-after studies (250 participants) showed a significant decrease in AHI . Four uncontrolled before-after studies (97 participants) with ODI4 as outcome also showed a significant decrease in oxygen desaturation index of 4% (ODI4) (-18.91 episodes/h [95% confidence interval -23.40, -14.43]).Conclusions: Published evidence suggests that weight loss through lifestyle and dietary interventions results in improvements in obstructive sleep apnea parameters, but is insufficient to normalize them. The changes in obstructive sleep apnea parameters could, however, be clinically relevant in some patients by reducing obstructive sleep apnea severity. |
| Whole-food plant-based diet reduces daytime sleepiness in patients with OSA. | Patel K, Lawson M, Cheung J. | 2023 | *Sleep Med*. 2023;107:327-329. doi:10.1016/j.sleep.2023.05.007 | Consumption of a diet high in saturated fat has been associated with daytime sleepiness. A whole-food plant-based (WFPB) dietary pattern, which is low in saturated fat, has been shown to be beneficial in a variety of health conditions. We assessed the effect of a short-term (21 days) WFPB diet intervention on daytime sleepiness in 14 patients with obstructive sleep apnea (OSA). We found a mean decrease of 3.8 points (SD = 3.3, p = 0.003) on the Epworth Sleepiness Scale (ESS) after switching from a standard Western diet to a WFPB diet. Our results suggest that a WFPB diet could be a viable dietary intervention to reduce symptom of daytime sleepiness. |
| Implementation of a Worksite Based 9-Week Micronutrient Dense Nutrition Intervention on Measures of Well-Being in a Cohort of Employees | Jay Sutliffe1,*, Julia Scheid1, Michelle Gorman2, Mary Jo Carnot3, Alison Adams4, Wendy Wetzel1, Tricia  Fortin5, Chloe Sutliffe6 & Joel Fuhrman7 | 2018 | Journal of Food and Nutrition, 4, 1-5. | This intervention has proven to be effective at improving employee well-being. Widespread workplace implementation should be considered to improve the overall wellness of employees and increased work productivity, which may help mitigate the increasingly demanding work environment many employees experience daily. |
| A Worksite Nutrition Intervention is Effective at Improving Employee Well-Being: A Pilot Study. | Sutliffe JT, Carnot MJ, Fuhrman JH, Sutliffe CA, Scheid JC | 2018 | *J Nutr Metab*. 2018;2018:8187203. Published 2018 May 2. doi:10.1155/2018/8187203 | Thirty-five university employees participated in a 6-week nutrition intervention. The dietary protocol emphasized the daily consumption of greens, beans/legumes, a variety of other vegetables, fruits, nuts, seeds, and whole grains, referred to as a micronutrient-dense, plant-rich diet. Participants were encouraged to minimize the consumption of refined foods and animal products. Results: Significant improvements in sleep quality, quality of life, and depressive symptoms were found. Conclusions: Findings reveal that a worksite nutrition intervention is effective at improving sleep quality, quality of life, and depressive symptoms with a projected improvement in work productivity and attendance. |

Supplemental Table 2.

| Document title | Authors | Year | Source | Results, conclusions |
| --- | --- | --- | --- | --- |
| Greater whole-grain intake is associated with lower risk of type 2 diabetes, cardiovascular disease, and weight gain | Ye, E.Q., Chacko, S.A., Chou, E.L., Kugizaki, M., Liu, S. | 2012 | [Journal of Nutrition 142(7), pp. 1304-1313](https://academic.oup.com/jn/article/142/7/1304/4743493?login=true) | Those consuming 48-80 g whole grain/d (3-5 serving/d) had an ~26% lower risk of T2D [RR = 0.74 (95% CI: 0.69, 0.80)], ~21% lower risk of CVD [RR = 0.79 (95% CI: 0.74, 0.85)], and consistently less weight gain during 8-13 y (1.27 vs 1.64 kg; P = 0.001). |
| A Low-Fat Vegan Diet Improves Glycemic Control and Cardiovascular Risk Factors in a Randomized Clinical Trial in Individuals With Type 2 Diabetes | Neal D. Barnard, MD; Joshua Cohen, MD; David J.A. Jenkins, MD, PHD; Gabrielle Turner-McGrievy, MS, RD; Lise Gloede, RD, CDE; Brent Jaster, MD; Kim Seidl, MS, RD; Amber A. Green, RD; Stanley Talpers, MD | 2006 | [Diabetes Care 1 August 2006; 29 (8): 1777–1783.](https://diabetesjournals.org/care/article/29/8/1777/28693/A-Low-Fat-Vegan-Diet-Improves-Glycemic-Control-and) | Both a low-fat vegan diet and a diet based on ADA guidelines improved glycemic and lipid control in type 2 diabetic patients. These improvements were greater with a low-fat vegan diet. |
| A low-fat vegan diet and a conventional diabetes diet in the treatment of type 2 diabetes: a randomized, controlled, 74-wk clinical trial | Neal D Barnard, Joshua Cohen, David JA Jenkins, Gabrielle Turner-McGrievy, Lise Gloede, Amber Green, Hope Ferdowsian | 2009 | [The American Journal of Clinical Nutrition, Volume 89, Issue 5, May 2009, Pages 1588S–1596S](https://academic.oup.com/ajcn/article/89/5/1588S/4596944) | Low-fat vegetarian and vegan diets were associated with sustained reductions in weight and plasma lipid concentrations. In an analysis controlling for medication changes, a low-fat vegan diet appeared to improve glycemia and plasma lipids more than did conventional diabetes diet recommendations. |
| Fruit consumption and risk of type 2 diabetes: Results from three prospective longitudinal cohort studies | Muraki, I., Imamura, F., Manson, J.E., (...), Van Dam, R.M., Sun, Q. | 2013 | [BMJ (Online)](https://www.bmj.com/content/347/bmj.f5001)  [347(7923),f5001](https://www.bmj.com/content/347/bmj.f5001) | Our findings suggest the presence of heterogeneity in the associations between individual fruit consumption and risk of type 2 diabetes. Greater consumption of specific whole fruits, particularly blueberries, grapes, and apples, is significantly associated with a lower risk of type 2 diabetes, whereas greater consumption of fruit juice is associated with a higher risk. |
| Association of plasma biomarkers of fruit and vegetable intake with incident type 2 diabetes: EPIC-InterAct case-cohort study in eight European countries | Zheng, J.-S., Sharp, S.J., Imamura, F., (...), Forouhi, N.G., Wareham, N.J. | 2020 | [The BMJ](https://www.bmj.com/content/370/bmj.m2194)  [370,m2194](https://www.bmj.com/content/370/bmj.m2194) | Conclusions These findings indicate an inverse association between plasma vitamin C, carotenoids, and their composite biomarker score, and incident type 2 diabetes in different European countries. These biomarkers are objective indicators of fruit and vegetable consumption, and suggest that diets rich in even modestly higher fruit and vegetable consumption could help to prevent development of type 2 diabetes. |
| Intake of whole grain foods and risk of type 2 diabetes: Results from three prospective cohort studies | Hu, Y., Ding, M., Sampson, L., (...), Hu, F.B., Sun, Q. | 2020 | [The BMJ](https://www.bmj.com/content/370/bmj.m2206)  [370,m2206](https://www.bmj.com/content/370/bmj.m2206) | Higher consumption of total whole grains and several commonly eaten whole grain foods, including whole grain breakfast cereal, oatmeal, dark bread, brown rice, added bran, and wheat germ, was significantly associated with a lower risk of type 2 diabetes. These findings provide further support for the current recommendations of increasing whole grain consumption as part of a healthy diet for the prevention of type 2 diabetes. |
| Higher whole-grain intake is associated with lower risk of type 2 diabetes among middle-aged men and women: The Danish diet, cancer, and health cohort | Kyrø, C., Tjønneland, A., Overvad, K., Olsen, A., Landberg, R. | 2018 | [Journal of Nutrition](https://academic.oup.com/jn/article/148/9/1434/5054990?login=true)  [148(9), pp. 1434-1444](https://academic.oup.com/jn/article/148/9/1434/5054990?login=true) | Whole-grain intake was associated with an 11% and 7% lower risk of type 2 diabetes per whole-grain serving (16 g) per day for men and women, respectively [HR (95% CI)—men: 0.89 (0.87, 0.91); women: 0.93 (0.91, 0.96)]. Conclusions  In this cohort study, we found consistent associations between high whole-grain intake and lower risk of type 2 diabetes. Overall, an association was found for all different cereals and whole-grain products tested. |
| Whole grain intake and glycaemic control in healthy subjects: A systematic review and meta-analysis of randomized controlled trials | Marventano, S., Vetrani, C., Vitale, M., (...), Riccardi, G., Grosso, G. | 2017 | [Nutrients](https://www.mdpi.com/2072-6643/9/7/769)  [9(7),769](https://www.mdpi.com/2072-6643/9/7/769) | Results: The meta-analysis of the 14 studies testing the acute effects of WG foods showed significant reductions of the post-prandial values of the glucose iAUC (0–120 min) by −29.71 mmol min/L (95% CI: −43.57, −15.85 mmol min/L), the insulin iAUC (0–120 min) by −2.01 nmol min/L (95% CI: −2.88, −1.14 nmol min/L), and the maximal glucose and insulin response. In 16 medium- and long-term RCTs, effects of WG foods on fasting glucose and insulin and homeostatic model assessment-insulin resistance values were not significant. The consumption of WG foods is able to improve acutely the postprandial glucose and insulin homeostasis compared to similar refined foods in healthy subjects. Further research is needed to better understand the long-term effects and the biological mechanisms. |
| Plant-Based Dietary Patterns and Incidence of Type 2 Diabetes in US Men and Women: Results from Three Prospective Cohort Studies | Satija, A., Bhupathiraju, S.N., Rimm, E.B., (...), Sun, Q., Hu, F.B. | 2016 | [PLoS Medicine](https://journals.plos.org/plosmedicine/article?id=10.1371/journal.pmed.1002039)  [13(6),e1002039](https://journals.plos.org/plosmedicine/article?id=10.1371/journal.pmed.1002039) | Our study suggests that plant-based diets, especially when rich in high-quality plant foods, are associated with substantially lower risk of developing T2D. This supports current recommendations to shift to diets rich in healthy plant foods, with lower intake of less healthy plant and animal foods. We found that having a diet that emphasized plant foods and was low in animal foods was associated with a reduction of about 20% in the risk of diabetes.  Consumption of a plant-based diet that emphasized specifically healthy plant foods was associated with a larger decrease (34%) in diabetes risk, while consumption of a plant-based diet high in less healthy plant foods was associated with a 16% increased diabetes risk. Increasing intake of healthy plant foods while moderately reducing intake of some animal foods, especially red and processed meats, may be beneficial for diabetes prevention.  These findings support the newly released 2015–2020 Dietary Guidelines for Americans. |
| Effectiveness of plant-based diets in promoting well-being in the management of type 2 diabetes: A systematic review | Toumpanakis, A., Turnbull, T., Alba-Barba, I. | 2018 | [BMJ Open Diabetes Research and Care](https://drc.bmj.com/content/6/1/e000534)  [6(1),e000534](https://drc.bmj.com/content/6/1/e000534) | Plant-based diets were associated with significant improvement in emotional well-being, physical well-being, depression, quality of life, general health, HbA1c levels, weight, total cholesterol and low-density lipoprotein cholesterol, compared with several diabetic associations’ official guidelines and other comparator diets. Plant-based diets can significantly improve psychological health, quality of life, HbA1c levels and weight and therefore the management of diabetes. *by me: They also found that plant-based diets showed a potential to improve diabetic neuropathic pain and triglyceride levels in type 2 diabetes patients.* |
| Vegetarian diet improves insulin resistance and oxidative stress markers more than conventional diet in subjects with Type2 diabetes | Kahleova, H., Matoulek, M., Malinska, H., (...), Kahle, M., Pelikanova, T. | 2011 | [Diabetic Medicine](https://pubmed.ncbi.nlm.nih.gov/21480966/)  [28(5), pp. 549-559](https://pubmed.ncbi.nlm.nih.gov/21480966/) | We found that a calorie-restricted vegetarian diet increased insulin sensitivity, reduced volume of visceral fat and improved plasma concentrations of adipokines and oxidative stress markers more than a conventional diet in patients with Type 2 diabetes over 24 weeks. Forty-three per cent of participants in the experimental group and 5% of participants in the control group reduced diabetes medication (P < 0.001). Body weight decreased more in the experimental group than in the control group. Conclusions: A calorie-restricted vegetarian diet had greater capacity to improve insulin sensitivity compared with a conventional diabetic diet over 24 weeks. The greater loss of visceral fat and improvements in plasma concentrations of adipokines and oxidative stress markers with this diet may be responsible for the reduction of insulin resistance. The addition of exercise training further augmented the improved outcomes with the vegetarian diet. |
| Vegetarian diets and incidence of diabetes in the Adventist Health Study-2 | Tonstad, S., Stewart, K., Oda, K., (...), Herring, R.P., Fraser, G.E. | 2013 | [Nutrition, Metabolism and Cardiovascular Diseases](https://www.ncbi.nlm.nih.gov/pmc/articles/PMC3638849/)  [23(4), pp. 292-299](https://www.ncbi.nlm.nih.gov/pmc/articles/PMC3638849/) | Vegetarian diets (vegan, lacto ovo, semi-) were associated with a substantial and independent reduction in diabetes incidence. In Blacks the dimension of the protection associated with vegetarian diets was as great as the excess risk associated with Black ethnicity. |
| Weight gain over 5 y in 21,966 meat-eating, fish-eating, vegetarian, and vegan men and women in EPIC-Oxford | Rosell, M., Appleby, P., Spencer, E., Key, T. | 2006 | [International Journal of Obesity](https://www.nature.com/articles/0803305)  [30(9), pp. 1389-1396](https://www.nature.com/articles/0803305) | The mean annual weight gain was 389 (SD 884) g in men and 398 (SD 892) g in women. The differences between meat-eaters, fish-eaters, vegetarians and vegans in age-adjusted mean BMI at follow-up were similar to those seen at baseline. Multivariable-adjusted mean weight gain was somewhat smaller in vegans (284 g in men and 303 g in women, P<0.05 for both sexes) and fish-eaters (338 g, women only, P<0.001) compared with meat-eaters. Men and women who changed their diet in one or several steps in the direction meat-eater → fish-eater → vegetarian → vegan showed the smallest mean annual weight gain of 242 (95% CI 133–351) and 301 (95% CI 238–365) g, respectively. Conclusion: During 5 years follow-up, the mean annual weight gain in a health-conscious cohort in the UK was approximately 400 g. Small differences in weight gain were observed between meat-eaters, fish-eaters, vegetarians and vegans. Lowest weight gain was seen among those who, during follow-up, had changed to a diet containing fewer animal food. |
| A low-fat vegan diet elicits greater macronutrient changes, but is comparable in adherence and acceptability, compared with a more conventional diabetes diet among individuals with type 2 diabetes | Barnard, N.D., Gloede, L., Cohen, J., (...), Green, A.A., Ferdowsian, H. | 2009 | [Journal of the American Dietetic Association](https://www-scopus-com.rossuniversity.idm.oclc.org/redirect/linking.uri?targetURL=https%3a%2f%2fdoi.org%2f10.1016%2fj.jada.2008.10.049&locationID=2&categoryID=4&eid=2-s2.0-62149099610&issn=00028223&linkType=ViewAtPublisher&year=2009&origin=resultslist&dig=ff600c3b7f4a9b76f6f6b535009ad44c&recordRank=1)  [109(2), pp. 263-272](https://www-scopus-com.rossuniversity.idm.oclc.org/redirect/linking.uri?targetURL=https%3a%2f%2fdoi.org%2f10.1016%2fj.jada.2008.10.049&locationID=2&categoryID=4&eid=2-s2.0-62149099610&issn=00028223&linkType=ViewAtPublisher&year=2009&origin=resultslist&dig=ff600c3b7f4a9b76f6f6b535009ad44c&recordRank=1) | RESULTS: All participants completed the initial 22 weeks; 90% (45/50) of American Diabetes Association guidelines diet group and 86% (42/49) of the vegan diet group participants completed 74 weeks. Fat and cholesterol intake fell more and carbohydrate and fiber intake increased more in the vegan group. At 22 weeks, group-specific diet adherence criteria were met by 44% (22/50) of members of the American Diabetes Association diet group and 67% (33/49) of vegan-group participants (P=0.019); the American Diabetes Association guidelines diet group reported a greater increase in dietary restraint; this difference was not significant at 74 weeks. Both groups reported reduced hunger and reduced disinhibition. Questionnaire responses rated both diets as satisfactory, with no significant differences between groups, except for ease of preparation, for which the 22-week ratings marginally favored the American Diabetes Association guideline group. Cravings for fatty foods diminished more in the vegan group at 22 weeks, with no significant difference at 74 weeks. CONCLUSIONS: Despite its greater influence on macronutrient intake, a low-fat, vegan diet has an acceptability similar to that of a more conventional diabetes diet. Acceptability appears to be no barrier to its use in medical nutrition [therapy.](http://therapy.at/) |
| Type of vegetarian diet, body weight, and prevalence of type 2 diabetes | Tonstad, S., Butler, T., Yan, R., Fraser, G.E. | 2009 | [Diabetes Care](https://diabetesjournals.org/care/article/32/5/791/29593/Type-of-Vegetarian-Diet-Body-Weight-and-Prevalence)  [32(5), pp. 791-796](https://diabetesjournals.org/care/article/32/5/791/29593/Type-of-Vegetarian-Diet-Body-Weight-and-Prevalence) | RESULTS - Mean BMI was lowest in vegans (23.6 kg/m2) and incrementally higher in lactoovo vegetarians (25.7 kg/m2), pesco-vegetarians (26.3 kg/m2), semi-vegetarians (27.3 kg/m2), and nonvegetarians (28.8 kg/m 2). Prevalence of type 2 diabetes increased from 2.9% in vegans to 7.6% in nonvegetarians; the prevalence was intermediate in participants consuming lacto-ovo (3.2%), pesco (4.8%), or semi-vegetarian (6.1%) diets. CONCLUSIONS - The 5-unit BMI difference between vegans and nonvegetarians indicates a substantial potential of vegetarianism to protect against obesity. Increased conformity to vegetarian diets protected against risk of type 2 diabetes after lifestyle characteristics and BMI were taken into account. Pesco- and semi-vegetarian diets afforded intermediate protection. |
| CARDIOVASCULAR DISEASES AND PBD |  |  |  |  |
| Document title | Authors | Year | Source | Results, conclusions |
| Dietary intake of total, animal, and plant proteins and risk of all cause, cardiovascular, and cancer mortality: Systematic review and dose-response meta-analysis of prospective cohort studies | Naghshi, S., Sadeghi, O., Willett, W.C., Esmaillzadeh, A. | 2020 | [The BMJ](https://www.scopus.com/record/display.uri?eid=2-s2.0-85088474415&origin=resultslist&sort=r-f&src=s&st1=Dietary+carbohydrate+intake+and+mortality%3a+a+prospective+cohort+study+and+meta-analysis&nlo=&nlr=&nls=&sid=62e1fb308cd597e6a38530bc3112097d&sot=b&sdt=b&sl=102&s=TITLE-ABS-KEY%28Dietary+carbohydrate+intake+and+mortality%3a+a+prospective+cohort+study+and+meta-analysis%29&relpos=1&citeCnt=83&searchTerm=)  [370,m2412](https://www.scopus.com/record/display.uri?eid=2-s2.0-85088474415&origin=resultslist&sort=r-f&src=s&st1=Dietary+carbohydrate+intake+and+mortality%3a+a+prospective+cohort+study+and+meta-analysis&nlo=&nlr=&nls=&sid=62e1fb308cd597e6a38530bc3112097d&sot=b&sdt=b&sl=102&s=TITLE-ABS-KEY%28Dietary+carbohydrate+intake+and+mortality%3a+a+prospective+cohort+study+and+meta-analysis%29&relpos=1&citeCnt=83&searchTerm=) | Intake of total protein was associated with a lower risk of all cause mortality. Intake of plant protein was significantly associated with a lower risk of all cause mortality and cardiovascular disease mortality , but not with cancer mortality. Intake of total and animal protein was not significantly associated with risk of cardiovascular disease and cancer mortality. A dose-response analysis showed a significant inverse dose-response association between intake of plant protein and all cause mortality. An additional 3% energy from plant proteins a day was associated with a 5% lower risk of death from all causes. Conclusions Higher intake of total protein was associated with a lower risk of all cause mortality, and intake of plant protein was associated with a lower risk of all cause and cardiovascular disease mortality. Replacement of foods high in animal protein with plant protein sources could be associated with longevity. |
| Vegan dietary pattern for the primary and secondary prevention of cardiovascular diseases | Rees K, Al-Khudairy L, Takeda A, Stranges S | 2021 | [Cochrane Database of Systematic Reviews 2021, Issue 2. Art. No.: CD013501.](https://www-cochranelibrary-com.rossuniversity.idm.oclc.org/cdsr/doi/10.1002/14651858.CD013501.pub2/full) | The review concludes that there is no information currently about the eFects of a vegan diet on cardiovascular disease occurrence. For adults at increased risk of CVD (most with overweight, obesity, or type 2 diabetes mellitus), moderate‐certainty evidence shows that a vegan dietary pattern focused on vegetables, fruits, grains, legumes, and foods with a low glycemic index and low in fats probably slightly reduces total cholesterol and LDL cholesterol (by 0.24 and 0.22 mmol/L, respectively) but probably also reduces HDL cholesterol (by 0.08 mmol/L) and increases triglycerides (by 0.11 mmol/L). Low‐certainty evidence suggests minimal to no important differences between groups in systolic and diastolic blood pressure. One small trial (65 participants) reported no serious adverse events associated with the vegan diet, and no trial assessed CVD mortality, myocardial infarction, or stroke. |
| Whole grain cereals for the primary or secondary prevention of cardiovascular disease | Sarah AM Kelly1, Louise Hartley2, Emma Loveman3, Jill L Colquitt3, Helen M Jones4, Lena Al-Khudairy4, Christine Clar5, Roberta Germanò6, Hannah R Lunn4, Gary Frost7, Karen Rees8 | 2017 | [Cochrane Database of Systematic Reviews 2017, Issue 8. Art. No.: CD005051.](https://www-cochranelibrary-com.rossuniversity.idm.oclc.org/cdsr/doi/10.1002/14651858.CD005051.pub3/full) | There is insufficient evidence from RCTs of an effect of whole grain diets on cardiovascular outcomes or on major CVD risk factors such as blood lipids and blood pressure. Trials were at unclear or high risk of bias with small sample sizes and relatively short‐term interventions, and the overall quality of the evidence was low. There is a need for well‐designed, adequately powered RCTs with longer durations assessing cardiovascular events as well as cardiovascular risk factors. |
| Markers of cardiovascular risk are not changed by increased whole-grain intake: the WHOLEheart study, a randomised, controlled dietary intervention | Iain A. Brownlee,1 Carmel Moore,2 Mark Chatfield,2 David P. Richardson,3 Peter Ashby,4 Sharron A. Kuznesof,1 Susan A. Jebb,2 and Chris J. Seal1,* | 2010 | [British Journal of Nutrition , Volume 104 , Issue 1 , 14 July 2010 , pp. 125 - 134](https://www.cambridge.org/core/journals/british-journal-of-nutrition/article/markers-of-cardiovascular-risk-are-not-changed-by-increased-wholegrain-intake-the-wholeheart-study-a-randomised-controlled-dietary-intervention/573BEC40AD8CD79E6E541914ADCBA98C) | Although reported WG intake was significantly increased among intervention groups, and demonstrated good participant compliance, there were no significant differences in any markers of CVD risk between groups. A period of 4 months may be insufficient to change the lifelong disease trajectory associated with CVD. |
| Vegetarian Diets and Blood Pressure A Meta-analysis | Yoko Yokoyama, Kunihiro Nishimura, Neal D. Barnard | 2014 | [JAMA Intern Med. 2014;174(4):577-587](https://jamanetwork.com/journals/jamainternalmedicine/article-abstract/1832195) | Consumption of vegetarian diets is associated with lower BP. Such diets could be a useful nonpharmacologic means for reducing BP. |
| A plant-based diet, Atherogenesis, and coronary artery diseae prevention | P Tuso, S Stoll, W W Li | 2015 | [Perm J. 2015 Winter; 19(1): 62–67.](https://www.ncbi.nlm.nih.gov/pmc/articles/PMC4315380/) | Elevating levels of blood polyphenols and lowering blood trimethylamine-N-oxide (TMAO) levels by eating a plant-based diet may promote health of vascular endothelial cell and prevent atherosclerotic CADs by at least three different proposed mechanisms. |
| Plant-based foods and prevention of cardiovascular disease: An overview | Hu, F.B. | 2003 | [American Journal of Clinical Nutrition 78(3 SUPPL.), pp. 544S-551S](https://academic.oup.com/ajcn/article/78/3/544S/4689995?login=true) | Evidence from prospective cohort studies indicates that a high consumption of plant-based foods such as fruit and vegetables, nuts, and whole grains is associated with a significantly lower risk of coronary artery disease and stroke. |
| Nutritional update for physicians: plant-based diets. | Tuso, P.J., Ismail, M.H., Ha, B.P., Bartolotto, C. | 2013 | [The Permanente journal 17(2), pp. 61-66](https://www.thepermanentejournal.org/doi/10.7812/TPP/12-085) | Healthy eating may be best achieved with a plant-based diet,. We present a case study as an example of the potential health benefits of such a diet. Research shows that plant-based diets are cost-effective, low-risk interventions that may lower body mass index, blood pressure, HbA1C, and cholesterol levels. They may also reduce the number of medications needed to treat chronic diseases and lower ischemic heart disease mortality rates. Physicians should consider recommending a plant-based diet to all their patients, especially those with high blood pressure, diabetes, cardiovascular disease, or obesity. |
| Plant-based diets and cardiovascular health | Satija, A., Hu, F.B. | 2018 | [Trends in Cardiovascular Medicine 28(7), pp. 437-441](https://www-sciencedirect-com.rossuniversity.idm.oclc.org/science/article/abs/pii/S1050173818300240?via%3Dihub) | This review summarizes the current evidence base examining the associations of plant-based diets with cardiovascular endpoints, and discusses the potential biological mechanisms underlying their health effects, practical recommendations and applications of this research, and directions for future research. Healthful plant-based diets should be recommended as an environmentally sustainable dietary option for improved cardiovascular health. |
| Plant protein and animal proteins: Do they differentially affect cardiovascular disease risk? | Richter, C.K., Skulas-Ray, A.C., Champagne, C.M., Kris-Etherton, P.M. | 2015 | [Advances in Nutrition 6(6), pp. 712-728](https://academic.oup.com/advances/article/6/6/712/4555152?login=true) | The potential mechanisms responsible for any specific effects of plant and animal protein are similarly multifaceted and include the amino acid content of particular foods, contributions from other nonprotein compounds provided concomitantly by the whole food, and interactions with the gut microbiome. Evidence to date is inconclusive, and additional studies are needed to further advance our understanding of the complexity of plant protein vs. animal protein comparisons. Nonetheless, current evidence supports the idea that CVD risk can be reduced by a dietary pattern that provides more plant sources of protein compared with the typical American diet and also includes animal-based protein foods that are unprocessed and low in saturated fat. |
| Whole grain consumption and risk of cardiovascular disease, cancer, and all cause and cause specific mortality: Systematic review and dose-response meta-analysis of prospective studies | Aune, D., Keum, N., Giovannucci, E., (...), Riboli, E., Norat, T. | 2016 | [BMJ (Online)](https://www.bmj.com/content/353/bmj.i2716)  [353,i2716](https://www.bmj.com/content/353/bmj.i2716) | Conclusions This meta-analysis provides further evidence that whole grain intake is associated with a reduced risk of coronary heart disease, cardiovascular disease, and total cancer, and mortality from all causes, respiratory diseases, infectious diseases, diabetes, and all non-cardiovascular, non-cancer causes. |
| Total and specific fruit and vegetable consumption and risk of stroke: A prospective study | Larsson, S.C., Virtamo, J., Wolk, A. | 2013 | [Atherosclerosis](https://pubmed.ncbi.nlm.nih.gov/23294925/)  [227(1), pp. 147-152](https://pubmed.ncbi.nlm.nih.gov/23294925/) | This study shows an inverse association of fruit and vegetable consumption with stroke risk. Particularly consumption of apples and pears and green leafy vegetables was inversely associated with stroke |
| Fruits and vegetables consumption and risk of stroke: A meta-analysis of prospective cohort studies | Hu, D., Huang, J., Wang, Y., Zhang, D., Qu, Y. | 2014 | [Stroke](https://www.ahajournals.org/doi/10.1161/STROKEAHA.114.004836)  [45(6), pp. 1613-1619](https://www.ahajournals.org/doi/10.1161/STROKEAHA.114.004836) | Fruits and vegetables consumption are inversely associated with the risk of stroke |
| Fruit, vegetable, and legume intake, and cardiovascular disease and deaths in 18 countries (PURE): a prospective cohort study | Miller, V., Mente, A., Dehghan, M., (...), Machiweni, T., Mapanga, R. | 2017 | [The Lancet](https://www-scopus-com.rossuniversity.idm.oclc.org/record/display.uri?eid=2-s2.0-85028451132&origin=resultslist&sort=r-f&src=s&st1=Total+and+specific+fruit+and+vegetable+consumption+and+risk+of+stroke%3a+a+prospective+study&nlo=&nlr=&nls=&sid=78d4669ab8b2dc045a6458b063a7d3b7&sot=b&sdt=b&sl=105&s=TITLE-ABS-KEY%28Total+and+specific+fruit+and+vegetable+consumption+and+risk+of+stroke%3a+a+prospective+study%29&relpos=3&citeCnt=315&searchTerm=)  [390(10107), pp. 2037-2049](https://www-scopus-com.rossuniversity.idm.oclc.org/record/display.uri?eid=2-s2.0-85028451132&origin=resultslist&sort=r-f&src=s&st1=Total+and+specific+fruit+and+vegetable+consumption+and+risk+of+stroke%3a+a+prospective+study&nlo=&nlr=&nls=&sid=78d4669ab8b2dc045a6458b063a7d3b7&sot=b&sdt=b&sl=105&s=TITLE-ABS-KEY%28Total+and+specific+fruit+and+vegetable+consumption+and+risk+of+stroke%3a+a+prospective+study%29&relpos=3&citeCnt=315&searchTerm=) | Fruit intake was associated with lower risk of cardiovascular, non-cardiovascular, and total mortality, while legume intake was inversely associated with non-cardiovascular death and total mortality (in fully adjusted models). For vegetables, raw vegetable intake was strongly associated with a lower risk of total mortality, whereas cooked vegetable intake showed a modest benefit against mortality. Interpretation Higher fruit, vegetable, and legume consumption was associated with a lower risk of non-cardiovascular, and total mortality. Benefits appear to be maximum for both non-cardiovascular mortality and total mortality at three to four servings per day (equivalent to 375–500 g/day). |
| New perspectives on dairy and cardiovascular health | Lovegrove, J.A., Hobbs, D.A. | 2016 | [Proceedings of the Nutrition Society](https://www.cambridge.org/core/journals/proceedings-of-the-nutrition-society/article/new-perspectives-on-dairy-and-cardiovascular-health/86E6F97F1EA771E69D4CD749EEB48B2F)  [75(3), pp. 247-258](https://www.cambridge.org/core/journals/proceedings-of-the-nutrition-society/article/new-perspectives-on-dairy-and-cardiovascular-health/86E6F97F1EA771E69D4CD749EEB48B2F) | These apparent benefits of milk and dairy foods have been attributed to their unique nutritional composition, and suggest that the elimination of milk and dairy may not be the optimum strategy for CVD risk reduction. |
| Consumption of Meat, Fish, Dairy Products, and Eggs and Risk of Ischemic Heart Disease | Key, T.J., Appleby, P.N., Bradbury, K.E., (...), Riboli, E., Danesh, J. | 2019 | [Circulation](https://www.ahajournals.org/doi/10.1161/CIRCULATIONAHA.118.038813)  [139(25), pp. 2835-2845](https://www.ahajournals.org/doi/10.1161/CIRCULATIONAHA.118.038813) | Conclusions: Risk for ischemic heart disease (IHD) was positively associated with consumption of red and processed meat and inversely associated with consumption of yogurt, cheese, and eggs, although the associations with yogurt and eggs may be influenced by reverse causation bias. It is not clear whether the associations with red and processed meat and cheese reflect causality, but they were consistent with the associations of these foods with plasma non-high-density lipoprotein cholesterol and for red and processed meat with systolic blood pressure, which could mediate such effects. |
| Red meat intake and risk of coronary heart disease among US men: Prospective cohort study | Al-Shaar, L., Satija, A., Wang, D.D., (...), Hu, F.B., Willett, W.C. | 2020 | [The BMJ](https://www.bmj.com/content/371/bmj.m4141)  [371,m4141](https://www.bmj.com/content/371/bmj.m4141) | total, unprocessed, and processed red meat intake were each associated with a modestly higher risk of CHD. Compared with red meat, the intake of one serving per day of combined plant protein sources (nuts, legumes, and soy) was associated with a lower risk of CHD compared with total red meat, 0.87 (0.79 to 0.95) compared with unprocessed red meat, and 0.83 (0.76 to 0.91) compared with processed red meat). Substitutions of whole grains and dairy products for total red meat and eggs for processed red meat were also associated with lower CHD risk. Conclusions: Substituting high quality plant foods such as legumes, nuts, or soy for red meat might reduce the risk of CHD. Substituting whole grains and dairy products for total red meat, and eggs for processed red meat, might also reduce this risk |
| Effects of red meat, white meat, and nonmeat protein sources on atherogenic lipoprotein measures in the context of low compared with high saturated fat intake: a randomized controlled trial | Bergeron, N., Chiu, S., Williams, P.T., M King, S., Krauss, R.M. | 2019 | [American Journal of Clinical Nutrition](https://academic.oup.com/ajcn/article/110/1/24/5494812)  [110(1),nzq035, pp. 24-33](https://academic.oup.com/ajcn/article/110/1/24/5494812) | Results: Analysis included participants who completed all 3 dietary protein assignments (61 for high saturated fatty acids (SFA); 52 for low SFA). LDL cholesterol and apoB were higher with red and white meat than with nonmeat, independent of SFA content for all, except apoB: red meat compared with nonmeat). This was due primarily to increases in large LDL particles, whereas small + medium LDL and total/high-density lipoprotein cholesterol were unaffected by protein source . Primary outcomes did not differ significantly between red and white meat. Independent of protein source, high compared with low SFA increased LDL cholesterol (P = 0.0003), apoB (P = 0.0002), and large LDL (P = 0.0002).Conclusions: The findings are in keeping with recommendations promoting diets with a high proportion of plant-based food but, based on lipid and lipoprotein effects, do not provide evidence for choosing white over red meat for reducing CVD risk. This trial was registered at Clinicaltrials.gov as NCT01427855. |
| Ultra-processed food intake and risk of cardiovascular disease: Prospective cohort study (NutriNet-Santé) | Srour, B., Fezeu, L.K., Kesse-Guyot, E., (...), Julia, C., Touvier, M. | 2019 | [The BMJ](https://www.bmj.com/content/365/bmj.l1451)  [365,l1451](https://www.bmj.com/content/365/bmj.l1451) | Results: During a median follow-up of 5.2 years, intake of ultra-processed food was associated with a higher risk of overall cardiovascular disease (1409 cases; hazard ratio for an absolute increment of 10 in the percentage of ultra-processed foods in the diet 1.12 (95% confidence interval 1.05 to 1.20); P<0.001, 518 208 person years, incidence rates in high consumers of ultra-processed foods (fourth quarter) 277 per 100 000 person years, and in low consumers (first quarter) 242 per 100 000 person years), coronary heart disease risk (665 cases; hazard ratio 1.13 (1.02 to 1.24); P=0.02, 520 319 person years, incidence rates 124 and 109 per 100 000 person years, in the high and low consumers, respectively), and cerebrovascular disease risk (829 cases; hazard ratio 1.11 (1.01 to 1.21); P=0.02, 520 023 person years, incidence rates 163 and 144 per 100 000 person years, in high and low consumers, respectively). |
| Risk of hospitalization or death from ischemic heart disease among British vegetarians and nonvegetarians: Results from the EPIC-Oxford cohort study1-3 | Crowe, F.L., Appleby, P.N., Travis, R.C., Key, T.J. | 2013 | [American Journal of Clinical Nutrition](https://academic.oup.com/ajcn/article/97/3/597/4571519?login=true)  [97(3), pp. 597-603](https://academic.oup.com/ajcn/article/97/3/597/4571519?login=true) | Conclusion: Consuming a vegetarian diet was associated with lower IHD risk, a finding that is probably mediated by differences in non-HDL cholesterol, and systolic blood pressure. |
| Benefits of a Low-Fat Plant-Based Diet | Dunn-Emke, Weidner, Dean Ornish | 2001 | [Obesity research, 9(11), 731-731](https://www.proquest.com/openview/bb7938b763aea6c2b888ce5f47f122f7/1?cbl=105348&pq-origsite=gscholar) | Plant-based diet not only contributes to weight loss and the prevention of many chronic diseases, but also results in plasma cholesterol lowering of the same magnitude as achieved by statin drugs, a 40% reduction in low-density lipoprotein cholesterol after 1 year, and the reversal of heart disease |

| Document title | Authors | Year | Source | Results, conclusions |
| --- | --- | --- | --- | --- |
| Whole-grain intake and mortality from all causes, cardiovascular disease, and cancer: A systematic review and dose-response meta-analysis of prospective cohort studies | Benisi-Kohansal, S., Saneei, P., Salehi-Marzijarani, M., Larijani, B., Esmaillzadeh, A. | 2016 | [Advances in Nutrition](https://academic.oup.com/advances/article/7/6/1052/4642973?login=true)  [7(6), pp. 1052-1065](https://academic.oup.com/advances/article/7/6/1052/4642973?login=true) | A greater intake of both total whole grains and specific whole-grain foods was significantly associated with a lower risk of all-cause mortality in the meta-analysis. The pooled RR for all-cause mortality for an increase of 3 servings total whole grains/d (90 g/d) was 0.83 (95% CI: 0.79, 0.88). Total whole-grain intake (0.84; 95% CI: 0.76, 0.93) and specific whole-grain foods (0.82; 95% CI: 0.75, 0.90) were also associated with a reduced risk of mortality from cardiovascular disease. Each additional 3 servings total whole grains/d was associated with a 25% lower risk of mortality from cardiovascular disease. An inverse association was observed between whole-grain intake and risk of mortality from total cancers (0.94; 95% CI: 0.91, 0.98). We found an inverse association between whole-grain intake and mortality from all causes, cardiovascular disease, and total cancers. |
| Dietary carbohydrate intake and mortality: a prospective cohort study and meta-analysis | Sara B Seidelmann, MD  Brian Claggett, PhD  Susan Cheng, MD  Mir Henglin, BA  Amil Shah, MD  Lyn M Steffen, PhD  et al. | 2018 | [The Lancet Public Health 3(9), pp. e419-e428](https://www.thelancet.com/journals/lanpub/article/PIIS2468-2667(18)30135-X/fulltext) | Both high and low percentages of carbohydrate diets were associated with increased mortality, with minimal risk observed at 50–55% carbohydrate intake. Low carbohydrate dietary patterns favouring animal-derived protein and fat sources, from sources such as lamb, beef, pork, and chicken, were associated with higher mortality, whereas those that favoured plant-derived protein and fat intake, from sources such as vegetables, nuts, peanut butter, and whole-grain breads, were associated with lower mortality, suggesting that the source of food notably modifies the association between carbohydrate intake and mortality. In the long term, high-fat, low-carb diets also increase the risk of heart disease and premature death from all causes. |
| Association between Soft Drink Consumption and Mortality in 10 European Countries | Mullee, A., Romaguera, D., Pearson-Stuttard, J., (...), Gunter, M.J., Murphy, N. | 2019 | [JAMA Internal Medicine](https://jamanetwork-com.rossuniversity.idm.oclc.org/journals/jamainternalmedicine/fullarticle/2749350)  [179(11), pp. 1479-1490](https://jamanetwork-com.rossuniversity.idm.oclc.org/journals/jamainternalmedicine/fullarticle/2749350) | Relevance: This study found that consumption of total, sugar-sweetened, and artificially sweetened soft drinks was positively associated with all-cause deaths in this large European cohort; the results are supportive of public health campaigns aimed at limiting the consumption of soft drinks. |
| Red meat and processed meat consumption and all-cause mortality: A meta-analysis | Larsson, S.C., Orsini, N. | 2014 | [American Journal of Epidemiology](https://academic.oup.com/aje/article/179/3/282/103471?login=true)  [179(3), pp. 282-289](https://academic.oup.com/aje/article/179/3/282/103471?login=true) | In a dose-response meta-analysis, consumption of processed meat and total red meat, but not unprocessed red meat, was statistically significantly positively associated with all-cause mortality in a nonlinear fashion. These results indicate that high consumption of red meat, especially processed meat, may increase all-cause mortality. |
| Vegetarian dietary patterns and mortality in adventist health study 2 | Orlich, M.J., Singh, P.N., Sabaté, J., Jaceldo-Siegl, K., Fan, J., Knutsen, S., Beeson, W.L., (...), Fraser, G.E. | 2013 | [JAMA Internal Medicine](https://jamanetwork-com.rossuniversity.idm.oclc.org/journals/jamainternalmedicine/fullarticle/1710093)  [173(13), pp. 1230-1238](https://jamanetwork-com.rossuniversity.idm.oclc.org/journals/jamainternalmedicine/fullarticle/1710093) | Significant associations with vegetarian diets were detected for cardiovascular mortality, noncardiovascular noncancer mortality, renal mortality, and endocrine mortality. CONCLUSIONS AND RELEVANCE: Vegetarian diets are associated with lower all-cause mortality and with some reductions in cause-specific mortality. Results appeared to be more robust in males. These favorable associations should be considered carefully by those offering dietary guidance. |

| OBESITY< WEIGHT CNANGES AND PBD | | | | |
| --- | --- | --- | --- | --- |
| Document title | Authors | Year | Source | Results, conclusions |
| Comparative effectiveness of plant-based diets for weight loss: A randomized controlled trial of five different diets | Gabrielle M.Turner-McGrievyPh.D., R.D.aCharis R.DavidsonM.P.H.aEllen E.WingardM.P.H., R.D.bSaraWilcoxPh.D.bEdward A.FrongilloPh.D.a | 2015 | [Nutrition](https://www.sciencedirect.com/science/article/pii/S0899900714004237?via%3Dihub)  [Volume 31, Issue 2, February 2015, Pages 350-358](https://www.sciencedirect.com/science/article/pii/S0899900714004237?via%3Dihub) | Vegan participants decreased their fat and saturated fat more than the pesco-vegetarian, semi-vegetarian, and omnivorous groups at both 2 and 6 mo (P < 0.05).Conclusions: Vegan diets may result in greater weight loss than more modest recommendations. |
| Less animal-based food, better weight status: Associations of the restriction of animal-based product intake with body-mass-index, depressive symptoms and personality in the general population | Medawar, E., Enzenbach, C., Roehr, S., (...), Riedel-Heller, S.G., Witte, A.V. | 2020 | [Nutrients](https://www.mdpi.com/2072-6643/12/5/1492)  [12(5),1492](https://www.mdpi.com/2072-6643/12/5/1492) | Higher restriction of animal-based product intake was associated with a lower BMI, but not with depression scores. Personality, i.e., lower extraversion, was related to higher frequency of animal product intake. Moreover, personality traits were significantly associated with depressive symptoms, i.e., higher neuroticism, lower extraversion, lower agreeableness, lower conscientiousness, and with higher BMI. These findings encourage future longitudinal studies to test the efficacy of restricting animal-based products as a preventive and therapeutic strategy for overweight and obesity. |
| A two-year randomized weight loss trial comparing a vegan diet to a more moderate low-fat diet | Turner-McGrievy, G.M., Barnard, N.D., Scialli, A.R. | 2007 | [Obesity](https://onlinelibrary.wiley.com/doi/full/10.1038/oby.2007.270)  [15(9), pp. 2276-2281](https://onlinelibrary.wiley.com/doi/full/10.1038/oby.2007.270) | Discussion: A vegan diet was associated with significantly greater weight loss than the NCEP diet at 1 and 2 years. Both group support and meeting attendance were associated with significant weight loss at follow-up. |
| Effect of a Low-Fat Vegan Diet on Body Weight, Insulin Sensitivity, Postprandial Metabolism, and Intramyocellular and Hepatocellular Lipid Levels in Overweight Adults: A Randomized Clinical Trial | Kahleova, H., Petersen, K.F., Shulman, G.I., (...), Holubkov, R., Barnard, N.D. | 2020 | [JAMA Network Open](https://jamanetwork-com.rossuniversity.idm.oclc.org/journals/jamanetworkopen/fullarticle/2773291)  [25454](https://jamanetwork-com.rossuniversity.idm.oclc.org/journals/jamanetworkopen/fullarticle/2773291) | Over the 16 weeks, body weight decreased in the intervention group by 5.9 kg. Thermic effect of food increased in the intervention group from baseline to 16 weeks and did not change significantly in the control group . The homeostasis model assessment index decreased) and PREDIM increasedin the intervention group. Hepatocellular lipid levels decreased in the intervention group by 34.4%, from a mean (SD) of 3.2% (2.9%) to 2.4% (2.2%), and intramyocellular lipid levels decreased by 10.4%, from a mean (SD) of 1.6 (1.1) to 1.5 (1.0) (P = .03). None of these variables changed significantly in the control group over the 16 weeks. The change in PREDIM correlated negatively with the change in body weight. Changes in hepatocellular and intramyocellular lipid levels correlated with changes in insulin resistance. Conclusions and Relevance A low-fat plant-based dietary intervention reduces body weight by reducing energy intake and increasing postprandial metabolism. The changes are associated with reductions in hepatocellular and intramyocellular fat and increased insulin sensitivity. |
| Low body mass index in non-meat eaters: The possible roles of animal fat, dietary fibre and alcohol | Appleby, P.N., Thorogood, M., Mann, J.I., Key, T.J. | 1998 | [International Journal of Obesity](https://www-nature-com.rossuniversity.idm.oclc.org/articles/0800607)  [22(5), pp. 454-460](https://www-nature-com.rossuniversity.idm.oclc.org/articles/0800607) | RESULTS: Mean BMI was lower in non-meat eaters than in meat eaters in all age groups for both men and women. Overall age-adjusted mean BMIs in kg/m2 were 23.18 and 22.05 for male meat eaters and non-meat eaters respectively (P<0.0001) and 22.32 and 21.32 for female meat eaters and non-meat eaters respectively (P<0.0001). In addition to meat consumption, dietary fibre intake, animal fat intake, social class and past smoking were all independently associated with BMI in both men and women; alcohol consumption was independently associated with BMI in men, and parity was independently associated with BMI in women. After adjusting for these factors, the differences in mean BMI between meat eaters and non-meat eaters were reduced by 36% in men and 31% in women. CONCLUSIONS: Non-meat eaters are thinner than meat eaters. This may be partly due to a higher intake of dietary fibre, a lower intake of animal fat, and only in men a lower intake of alcohol. |
| Risk of overweight and obesity among semivegetarian, lactovegetarian, and vegan women | P K Newby 1 , Katherine L Tucker, Alicja Wolk | 2005 | [Jun;81(6):1267-74. doi: 10.1093/ajcn/81.6.1267.](https://pubmed.ncbi.nlm.nih.gov/15941875/) | Conclusions Even if vegetarians consume some animal products, our results suggest that self-identified semivegetarian, lactovegetarian, and vegan women have a lower risk of overweight and obesity than do omnivorous women. The advice to consume more plant foods and less animal products may help individuals control their weight. |
| Weight gain over 5 years in 21 966 meat-eating, fish-eating, vegetarian, and vegan men and women in EPIC-Oxford | M Rosell, P Appleby, E Spencer & T Key | 2006 | [International Journal of Obesity volume 30, pages 1389–1396 (2006)](https://www.nature.com/articles/0803305) | During 5 years follow-up, the mean annual weight gain in a health-conscious cohort in the UK was approximately 400 g. Small differences in weight gain were observed between meat-eaters, fish-eaters, vegetarians and vegans. Lowest weight gain was seen among those who, during follow-up, had changed to a diet containing fewer animal food. |
| Dietary adherence and acceptability of five different diets, including vegan and vegetarian diets, for weight loss: The New DIETs study | Moore, W.J., McGrievy, M.E., Turner-McGrievy, G.M. | 2015 | [Eating Behaviors](https://www-scopus-com.rossuniversity.idm.oclc.org/redirect/linking.uri?targetURL=https%3a%2f%2fdoi.org%2f10.1016%2fj.eatbeh.2015.06.011&locationID=2&categoryID=4&eid=2-s2.0-84936741380&issn=14710153&linkType=ViewAtPublisher&year=2015&origin=resultslist&dig=b1d141dc8eef8fb3fa2b4e80a73ea8d3&recordRank=1)  [19, pp. 33-38](https://www-scopus-com.rossuniversity.idm.oclc.org/redirect/linking.uri?targetURL=https%3a%2f%2fdoi.org%2f10.1016%2fj.eatbeh.2015.06.011&locationID=2&categoryID=4&eid=2-s2.0-84936741380&issn=14710153&linkType=ViewAtPublisher&year=2015&origin=resultslist&dig=b1d141dc8eef8fb3fa2b4e80a73ea8d3&recordRank=1) | No differences were found in dietary adherence or changes in FAQ, TFEQ, or PFS among the groups. At six months, non-adherent vegan and vegetarian participants (n = 16) had a significantly greater decrease in cholesterol intake (- 190.2 ± 199.2 mg) than non-adherent pesco-vegetarian/semi-vegetarian (n = 15, - 2.3 ± 200.3 mg, P = 0.02) or omnivore participants (n = 7, 17.0 ± 36.0, P = 0.04). Non-adherent vegan/vegetarian participants lost significantly more weight at six months (- 6.0 ± 6.7%) than non-adherent omnivore participants (- 0.4 ± 0.6%, P = 0.04). Dietary preference had no impact on adherence at six months. Due to equal rates of adherence and acceptability among the diet groups, instructing participants to follow vegan or vegetarian diets may have a greater impact on weight loss and animal product intake than providing instruction in more moderate approaches even among non-adherent participants. |

| Document title | Authors | Year | Source | Results, conclusions |
| --- | --- | --- | --- | --- |
| Vegetarian, vegan diets and multiple health outcomes: A systematic review with meta-analysis of observational studies | Dinu, M., Abbate, R., Gensini, G.F., Casini, A., Sofi, F. | 2017 | [Critical Reviews in Food Science and Nutrition 57(17), pp. 3640-3649](https://www-tandfonline-com.rossuniversity.idm.oclc.org/doi/abs/10.1080/10408398.2016.1138447?journalCode=bfsn20) | This comprehensive meta-analysis reports a significant protective effect of a vegetarian diet versus the incidence and/or mortality from ischemic heart disease (−25%) and incidence from total cancer (−8%). Vegan diet conferred a significant reduced risk (−15%) of incidence from total cancer. |
| Mortality in vegetarians and nonvegetarians: Detailed findings from a collaborative analysis of 5 prospective studies | Key, T.J., Fraser, G.E., Thorogood, M., (...), Mann, J., McPherson, K. | 1999 | [American Journal of Clinical Nutrition](https://academic.oup.com/ajcn/article/70/3/516s/4714974?login=true)  [70(3 SUPPL.), pp. 516S-524S](https://academic.oup.com/ajcn/article/70/3/516s/4714974?login=true) | This pattern of putative risk factors would likely contribute to increases in both insulin resistance (high body mass, high red meat intake) and glycemic load (low legume intake), a synergism that, if causal, implicates hyperinsulinemic exposure in colon carcinogenesis. The overall findings from this cohort identify both red meat intake and white meat intake as important dietary risk factors for colon cancer and raise the possibility that the risk due to red meat intake reflects a more complex etiology |
| Cancer incidence in vegetarians: Results from the European Prospective Investigation into Cancer and Nutrition (EPIC-Oxford) | Key, T.J., Appleby, P.N., Spencer, E.A., (...), Roddam, A.W., Allen, N.E. | 2009 | [American Journal of Clinical Nutrition](https://academic.oup.com/ajcn/article/89/5/1620S/4596951?login=true)  [89(5), pp. 1620S-1626S](https://academic.oup.com/ajcn/article/89/5/1620S/4596951?login=true) | Conclusions: The overall cancer incidence rates of both the vegetarians and the nonvegetarians in this study are low compared with national rates. Within the study, the incidence of all cancers combined was lower among vegetarians than among meat eaters, but the incidence of colorectal cancer was higher in vegetarians than in meat eaters!. |
